# New Life of an Old Drug: Caffeine as a Modulator of Antibacterial Activity of Commonly Used Antibiotics

**DOI:** 10.3390/ph15070872

**Published:** 2022-07-15

**Authors:** Anna Woziwodzka, Marta Krychowiak-Maśnicka, Grzegorz Gołuński, Anna Łosiewska, Agnieszka Borowik, Dariusz Wyrzykowski, Jacek Piosik

**Affiliations:** 1 Laboratory of Biophysics, Intercollegiate Faculty of Biotechnology, University of Gdansk and Medical University of Gdansk, 80-307 Gdansk, Poland; grzegorz.golunski@ug.edu.pl (G.G.); anna.losiewska@blirt.eu (A.Ł.); agnieszka.borowik@gmail.com (A.B.); jacek.piosik@ug.edu.pl (J.P.); 2 Laboratory of Biologically Active Compounds, Intercollegiate Faculty of Biotechnology, University of Gdansk and Medical University of Gdansk, 80-307 Gdansk, Poland; marta.krychowiak@ug.edu.pl; 3 Aging and Metabolism Research Program, Oklahoma Medical Research Foundation OMRF, Oklahoma City, OK 73104, USA; 4 Department of Inorganic Biological Chemistry, Faculty of Chemistry, University of Gdansk, 80-308 Gdansk, Poland; dariusz.wyrzykowski@ug.edu.pl

**Keywords:** antibacterial agent, antimicrobial resistance, azithromycin, caffeine, cefepime, drug repositioning, gentamycin, novobiocin, synergy, ticarcillin

## Abstract

With the rapid and continuous emergence of antimicrobial resistance, bacterial infections became a significant global healthcare concern. One of the proposed strategies to combat multidrug-resistant pathogens is to use additional compounds, such as natural biologically active substances, as adjuvants for existing antibiotics. In this study, we investigated the potential of caffeine, the widely consumed alkaloid, to modulate the antibacterial effects of antibiotics commonly used in clinical practice. We used disc diffusion assay to evaluate the effects of caffeine on 40 antibiotics in two *Staphylococcus aureus* strains (methicillin-resistant and methicillin-sensitive). Based on the results of this step, we selected five antibiotics for which the greatest caffeine-induced improvements in antibacterial activity were observed, and further analyzed their interactions with caffeine using a checkerboard approach. Caffeine at concentrations of 250 µg/mL or higher halved the MIC values of ticarcillin, cefepime, gentamycin, azithromycin, and novobiocin for all gram-negative species investigated (*Pseudomonas aeruginosa*, *Klebsiella pneumoniae*, and *Acinetobacter baumannii*). At the highest caffeine concentrations tested (up to 16 mg/mL), decreases in MIC values were 8- to 16-fold. The obtained results prove that caffeine modulates the activity of structurally diverse antibiotics, with the most promising synergistic effects observed for cefepime and azithromycin toward gram-negative pathogens.

## 1. Introduction

Antimicrobial resistance represents one of the most important challenges to public health globally [1]. Overuse or misuse of antibiotics in clinical settings, as well as in agriculture to promote the growth of livestock animals, resulted in the emergence of antibiotic resistance and led to a significant reduction in antibiotic potential to effectively control infectious diseases [2,3]. Scientific and commercial experience from the last decades demonstrate that finding new antibiotics for clinical applications is not only challenging (with only a few successful examples of antibiotics that reached the market recently [4]), but also often counteracted by progressing antimicrobial resistance [5].

Apart from attempts to develop new chemical entities, with limited success to date [6], an alternative strategy is to repurpose existing drugs (including antibiotics and compounds with other indications) for treating bacterial infections. Such drug repositioning was successfully applied for thalidomide, which was initially used for morning sickness in the 1950s and later approved for multiple myeloma treatment in 2006 [7], and thioguanine, originally used for leukemia treatment and then as a rescue immunosuppressant in inflammatory bowel disease [8]. Reviving old antibiotics also proved effective in treating infectious diseases. Indeed, several compounds registered up to six decades ago, but abandoned due to their unfavorable safety profile or limited efficacy, were recently redeveloped and applied in clinical practice [9]. For example, colistin, which fell out of favor in the 1970s, is increasingly used as a last-line therapy in critically ill patients [10], as it retains significant activity against key gram-negative pathogens [11]. Thus, uncovering antimicrobial activities of drugs with other indications appears as a cost- and time-effective alternative to de novo antibiotic discovery and development [12].

Caffeine, due to its psychostimulant effects, is by far the most widely consumed alkaloid on a global scale [13]. Apart from its natural existence in the leaves, fruits, and seeds of a broad range of plants such as coffee, tea, or guarana, synthetic caffeine is used as an additive to soft drinks and energy drinks [14]. A typical single dose of caffeine related to its dietary consumption is approximately 50 mg, which results in its peak plasma concentration of up to 2 mg/L [15,16]. Caffeine is also a component of anti-fatigue tablets [14] and is used as a drug for treating apnea of prematurity in infants [17] and in combination with analgesics as a pain killer [18]. Thanks to its broad and long-term usage, the pharmacological and safety profile of caffeine is very well-established. Side effects of caffeine, usually associated with its high intake, include anxiety, restlessness, insomnia, and psychomotor agitation [19]. Toxic effects are associated with caffeine intake of 1.2 g or higher, whereas doses above 10 g are considered fatal [20]. The recommended daily dose of caffeine for non-pregnant adults is up to 400 mg [21]. Although isolated caffeine intake results in a moderate increase in blood pressure (the effect is observed in the short term only, with tolerance usually developing within a week) [22], daily consumption of three to five cups of caffeinated coffee is associated with a reduced risk of cardiovascular diseases [23]. Moreover, epidemiologic and worldwide cohort studies showed that caffeine can reduce the risk of depression, suicide, Parkinson’s disease, liver fibrosis, cirrhosis, and several cancer types (e.g., melanoma and nonmelanoma skin cancer, breast and prostate cancer, and hepatocellular carcinoma) [24]. Recently, following a drug repurposing approach, caffeine was proposed for treatment of COVID-19 infections [25,26] and was identified with therapeutic effects on hypertension biomarkers [27].

This study investigates the in vitro potential of caffeine to enhance the antibacterial effects of well-established antibiotics routinely used in clinical practice. First, we evaluated the modulatory effects of caffeine on a total of 30 antibiotics representing diverse antibiotic classes and various mechanisms of action against two *Staphylococcus aureus* strains. The majority of the antibiotics tested belong to the WHO List of Essential Medicines [28]. Next, we selected five of the most promising antibiotic-caffeine pairs (for which the most pronounced improvements were observed in the screening phase) and tested their antibacterial potential towards a panel of six important human pathogens with diverse levels of antibiotic resistance. Finally, to gain insight into the putative mechanisms behind the modulatory effects, we evaluated the possibility of direct interactions between the antibiotics and caffeine.

## 2. Results

### 2.1. Antibacterial Screening

**Figure 1:**
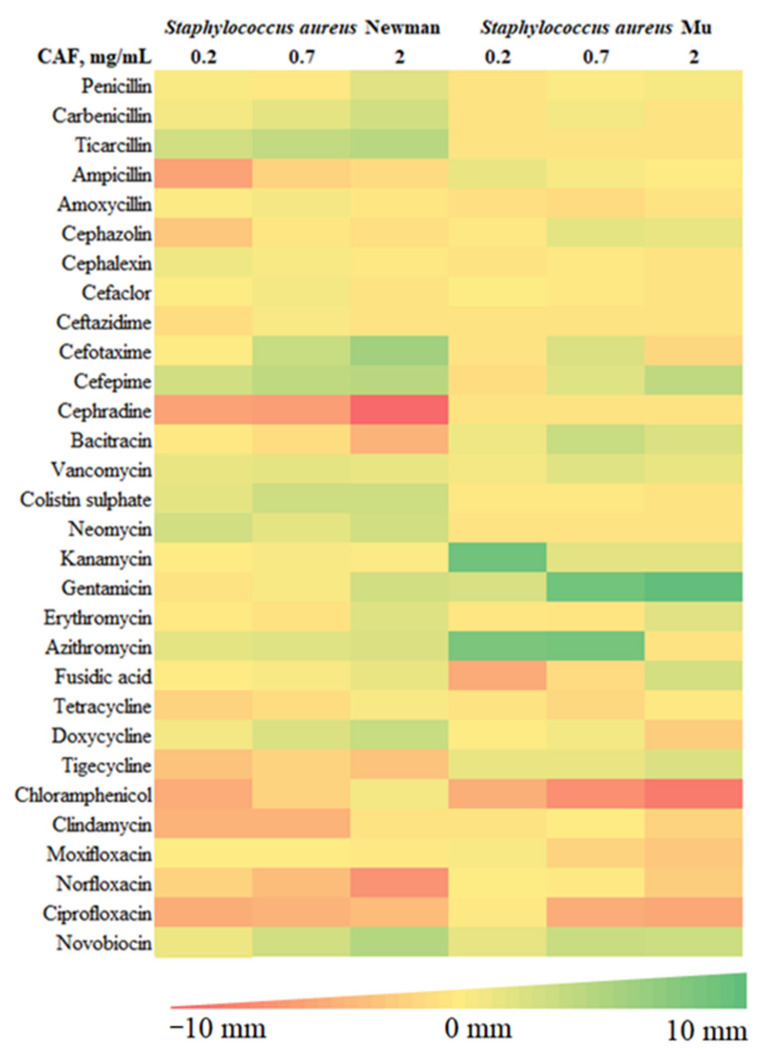
Graphical representation of disc diffusion assay screening. Caffeine (CAF)–antibiotic interactions were measured using a disc diffusion assay with agar growth medium supplemented with indicated concentrations of caffeine. Colors correspond to mean differences in the growth inhibition diameters compared with plates without caffeine.

To evaluate the impact of caffeine against a broad range of antibiotics, we used a modified disc diffusion susceptibility assay on two *S. aureus* strains (Newman and Mu) with a diverse pattern of antibiotic susceptibility. Possible inhibition or enhancement of antibiotic activity by caffeine was expressed as the difference in the diameter of bacterial growth inhibition around the discs placed on the agar with and without caffeine The summary of results of the disc diffusion test for 30 antibiotics representing various mechanisms of action are shown in Table 1. For a graphical representation of the screening phase, refer to Figure 1.

Modulatory effects of caffeine were observed for a broad range of antibiotics against both strains of *S. aureus*. The observed pattern of caffeine impact was generally consistent within particular antibiotic families. For penicillins, no relevant impact of caffeine was reported in *S. aureus* antibiotic-resistant strain Mu. For penicillin and carboxypenicillins (carbenicillin and ticarcillin), a slight caffeine-induced enhancement of antibacterial activity was observed in *S. aureus* Newman strain, with no such effects in ampicillins. For most cephalosporins investigated, weak inhibitory effects of caffeine were observed against both strains of *S. aureus*. Strong inhibition of caffeine was reported only in combination with cephradine against *S. aureus* Newman strain. In contrast, the activity of third- and fourth-generation cephalosporins, cefotaxime and cefepime, increased in a moderate manner, particularly in *S. aureus* Newman strain. No notable impact of caffeine was observed for nonribosomal peptide antibiotics bacitracin, vancomycin, and colistin. For aminoglycosides (neomycin, kanamycin, and gentamycin), macrolides (in particular azithromycin), and aminocoumarin novobiocin, a pronounced potentiation of antistaphylococcal activity was observed in the presence of caffeine. No marked modulatory action of caffeine was reported toward fusidic acid and tetracyclines. In contrast, caffeine considerably inhibited the antibacterial activity of chloramphenicol, clindamycin, and fluoroquinolones moxifloxacin, norfloxacin, and ciprofloxacin.

Summarizing, a marked enhancement of antistaphylococcal activity in the presence of caffeine was observed for five antibiotics: novobiocin, cefepime, gentamycin, azithromycin, and ticarcillin. As a result of the screening phase, these antibiotics were selected to be further tested along with caffeine against diverse pathogenic bacterial species.

### 2.2. Effects of Caffeine on Antibacterial Activity of Selected Antibiotics

Five antibiotics, novobiocin, cefepime, gentamycin, azithromycin, and ticarcillin, representing diverse structures and antibacterial mechanisms, were selected for a detailed evaluation with respect to possible modulatory effects of caffeine on different bacterial species. The selection was based on the findings of the screening phase of the study; antibiotics with the most pronounced caffeine-induced potentiation of activity were selected.

To evaluate the modulatory potential of caffeine toward selected antibiotics in more detail, we used a broth microdilution method along with a checkerboard technique. This allowed for assessing the type of interaction between the two compounds when used simultaneously against human pathogenic bacteria. We used two strains of gram-positive *Staphylococcus aureus* (MSSA ATCC 25923 and MRSA 43300), and three strains of gram-negative bacteria (*Pseudomonas aeruginosa* ATCC 27853, *Acinetobacter baumannii* ATCC 19606, and *Klebsiella pneumoniae* ATCC 700603).

The antibacterial activity of caffeine itself varied across the pathogens evaluated. For *S. aureus* MSSA and MRSA, as well as for *P. aeruginosa*, antibacterial activity of caffeine was not observed up to its concentrations of 16 mg/mL. For *K. pneumoniae*, only weak antibacterial activity was reported with a MIC value of 16 mg/mL. The most pronounced antibacterial effect of caffeine was observed against *A. baumannii*, with a MIC value of 4 mg/mL.

**Figure 2:**
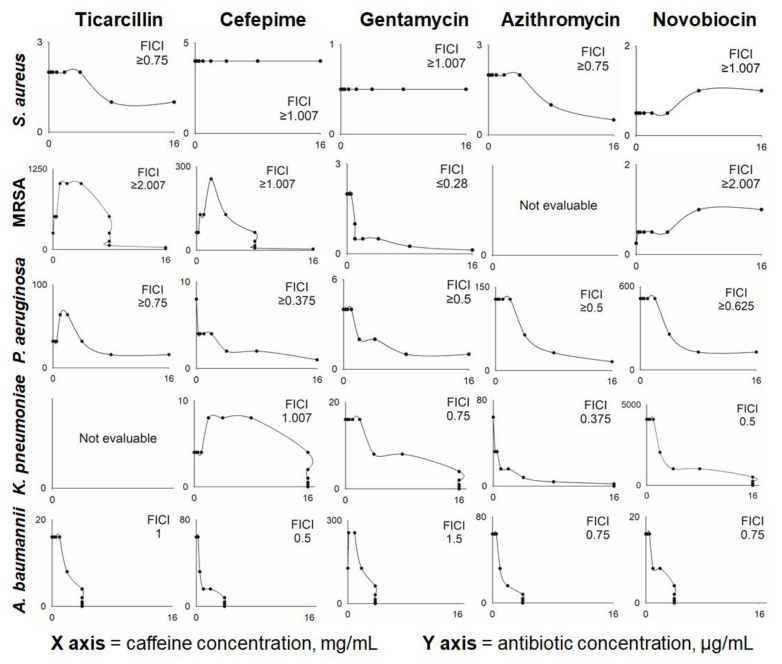
Dose-dependent modulatory properties of caffeine toward antibiotics (ticarcillin, cefepime, gentamycin, azithromycin, and novobiocin, each shown in a separate column) in selected bacterial pathogens. Graphs represent isobolograms for each antibiotic-caffeine pair tested in concentration gradient of both compounds. Five evaluated pathogens are given as separate rows. Inhibitory concentrations given on Y axes correspond to minimal inhibitory concentration of tested antibiotics. FICI, Fractional Inhibitory Concentration Index, calculated for each tested antibiotic-caffeine-pathogen combination according to Odds [29]. *S. aureus*, *Staphylococcus aureus* ATCC 25923; MRSA, *Staphylococcus aureus* ATCC MRSA 43300; *P. aeruginosa*, *Pseudomonas aeruginosa* ATCC 27853; *K. pneumoniae*, *Klebsiella pneumoniae* ATCC 700603; and *A. baumannii*, *Acinetobacter baumannii* ATCC 19606.

The results of the checkerboard experiments are shown in Table 2. For a graphical representation of obtained results, expressed as isobolograms, refer to Figure 2 (for full-size isobolograms, see Figures S1–S5). For the vast majority of antibiotic-caffeine combinations, an enhancement of antibacterial activity was reported regardless of the pathogen tested. Although additive and synergistic effects were observed most frequently, antagonistic interactions were also observed when caffeine was applied with the following antibiotics: i. ticarcillin towards *S. aureus* MRSA 43300 (at caffeine concentrations up to 8 mg/mL); ii. cefepime towards *S. aureus* MRSA 43300 and *K. pneumoniae* ATCC 700603; and iii. novobiocin towards *S. aureus* ATCC 27853 and MRSA 43300. No indication of interactions (neither positive nor negative) was found only for cefepime and gentamycin applied simultaneously with caffeine towards *S. aureus* ATCC 27853 (MSSA). In most cases, the addition of relatively low concentrations of caffeine (0.5 to 8 mg/mL) resulted in halving the MIC values of tested antibiotics. Caffeine at concentrations of 0.25 mg/mL caused a 2-fold drop in MIC value of cefepime towards *P. aeruginosa* and of azithromycin towards *K. pneumoniae*. Similarly, 0.5 mg/mL of caffeine halved the MIC of cefepime towards *A. baumannii*. Subinhibitory doses of caffeine led to as high as 16-fold decreases in MIC values of ticarcillin, gentamycin, and cefepime for *S. aureus* MRSA strain, azithromycin for *K. pneumoniae*, and 8-fold MIC decreases in cefepime and azithromycin for both *P. aeruginosa* and *A. baumannii*. Antagonistic effects of caffeine (with an increase in MIC of 2- to 4-fold) were reported for its combination with novobiocin for both *S. aureus* strains, with cefepime for *K. pneumoniae*, and with gentamycin for *A. baumannii*.

A dose-dependent interaction was observed between caffeine and ticarcillin for selected pathogens. Although lower concentrations of caffeine inhibited the antibiotic (up to a 4-fold increase in MIC), at higher caffeine doses antibacterial activity of ticarcillin increased, with a 4-fold decrease in MIC reported for *S. aureus* MRSA and a 2-fold decrease in MIC reported for *P. aeruginosa*. For both pathogens, no inhibitory effects of caffeine alone were reported in concentrations up to 16 mg/mL, thus indicating a probable synergistic interaction between ticarcillin and caffeine.

Overall, the most pronounced caffeine-induced potentiation of antibacterial activity was observed toward *S. aureus* MRSA strain, *P. aeruginosa*, and *A. baumannii*, whereas moderate effects on selected antibiotics were observed for *S. aureus* MSSA and *K. pneumoniae*. Among five antibiotics tested, the overall potential of caffeine to enhance antibacterial activity was the greatest for cefepime and azithromycin, and the weakest for novobiocin.

### 2.3. Antibacterial Activity of Caffeine against Clinical Isolates of Staphylococcus Aureus

Given the diverse modulatory activity of caffeine toward two evaluated strains of *S. aureus,* with more pronounced results observed for the *S. aureus* MRSA strain, we aimed to further investigate the antibacterial potential of caffeine itself against a broad range of clinical isolates of *S. aureus* representing various extents and patterns of antibiotic resistance. MIC values of caffeine ranged from 4 mg/mL to >16 mg/mL. The antibacterial activity of caffeine was overall greater for MRSA isolates than for MSSA isolates (Table 3). Although the number of isolates with the lowest MIC values (of 4 mg/mL) and the highest MIC values (>16 mg/mL) was comparable for MRSA and MSSA bacteria, the proportion of isolates moderately susceptible to caffeine (a MIC value of 8 mg/mL) was higher for MRSA than MSSA. For detailed characteristics of investigated isolates and obtained MIC values of caffeine, refer to Table S1.

Two isolates from the MRSA group and two MSSA isolates presented with the highest level of caffeine susceptibility (MIC values of 4 mg/mL). Three isolates were obtained from the nasal cavity. Two caffeine-susceptible MRSA isolates exhibited resistance to multiple antibiotics, i.e., high resistance to rifamycins, partial resistance or resistance to quinolones, resistance to fusidic acid, intermediate resistance to tetracyclines, and resistance to aminoglycosides (kanamycin, gentamycin, and tobramycin). One of the MSSA isolates was resistant to quinolones and partially resistant to tetracyclines, whereas the second MSSA isolate was in general susceptible to a broad range of antibiotics.

### 2.4. Molecular Interactions between Caffeine and Selected Antibiotics

To investigate direct interactions between caffeine and antibiotics, we used two complementary biophysical methods for studying interactions of small ligands in an aqueous solution: UV-Vis spectroscopy and isothermal titration calorimetry (ITC).

In UV-Vis spectroscopy, a solution of each of five antibiotics selected in the screening phase (i.e., ticarcillin, cefepime, gentamycin, azithromycin, and novobiocin) was titrated with increasing concentrations of caffeine solution, and respective UV-Vis spectra were recorded after each titration. All spectra were analyzed at wavelengths of >320 nm to avoid the background effects of caffeine absorption. The absorption range of ticarcillin, azithromycin, cefepime, and gentamycin fully overlapped with the absorption of caffeine, which rendered the analysis of 2-component mixture spectra impossible (for details, see Figure S6).

**Figure 3:**
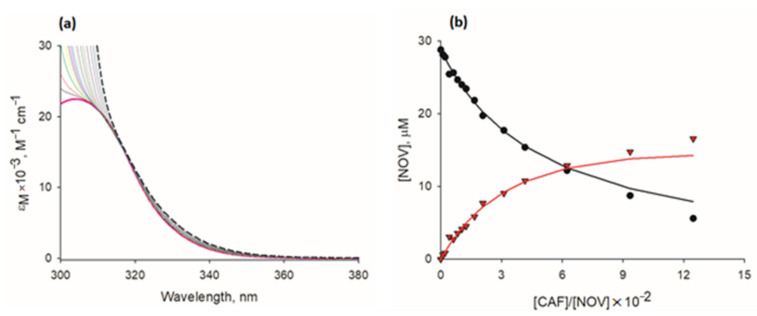
Spectrophotometric analysis of interactions of novobiocin with caffeine. (**a**) Molar extinction coefficient (ε_M_) spectra of novobiocin (initial concentration, 28.8 µM) titrated with caffeine (concentration range, 0.3–32.7 mM); spectrum of novobiocin in its free form is marked in bold red. Theoretical spectrum of novobiocin complexed with caffeine (determined by extrapolation of spectra of novobiocin-caffeine mixtures to the concentration ratio of novobiocin to caffeine tending to 0) is marked with black dashed lines. (**b**) Comparison of experimental and theoretical (model-based) concentrations in novobiocin-caffeine mixtures analyzed spectrophotometrically. Points represent concentrations of novobiocin in a free form (circles) and novobiocin in the complex with caffeine (triangles), calculated using two-component decomposition of mixture spectra. Lines represent concentrations of novobiocin in a free form (black line) and novobiocin in a complex with caffeine (red line), calculated using statistical-thermodynamical model of mixed aggregation [30] with K_AC_ ± standard error value of 30.51 M^−1^ ± 1.72 M^−1^. CAF, caffeine; NOV, novobiocin.

Absorption spectra normalized to the concentration of absorbing ligand (i.e., novobiocin) are shown in Figure 3a. Observed changes in the spectra of novobiocin upon addition of increasing concentrations of caffeine are related to a new absorbing component that appears in the mixture, thus providing evidence for direct interactions between studied ligands.

Based on spectra additivity law, we calculated molar fractions of free novobiocin and novobiocin complexed with caffeine in each novobiocin-caffeine mixture measured. To further analyze interactions in a quantitative manner, we employed a statistical-thermodynamical model of mixed aggregation (Zdunek et al. [30]), which allowed for calculating concentrations of all components in the novobiocin-caffeine mixture on each step of spectroscopic titration and estimating association constants of ligand aggregation. Experimental and theoretical (modelled) concentrations of free novobiocin and novobiocin complexed with caffeine are shown in Figure 3b. The association constant of novobiocin-caffeine interactions determined by spectroscopy combined with statistical-thermodynamical calculations is shown in Table 4.

**Figure 4:**
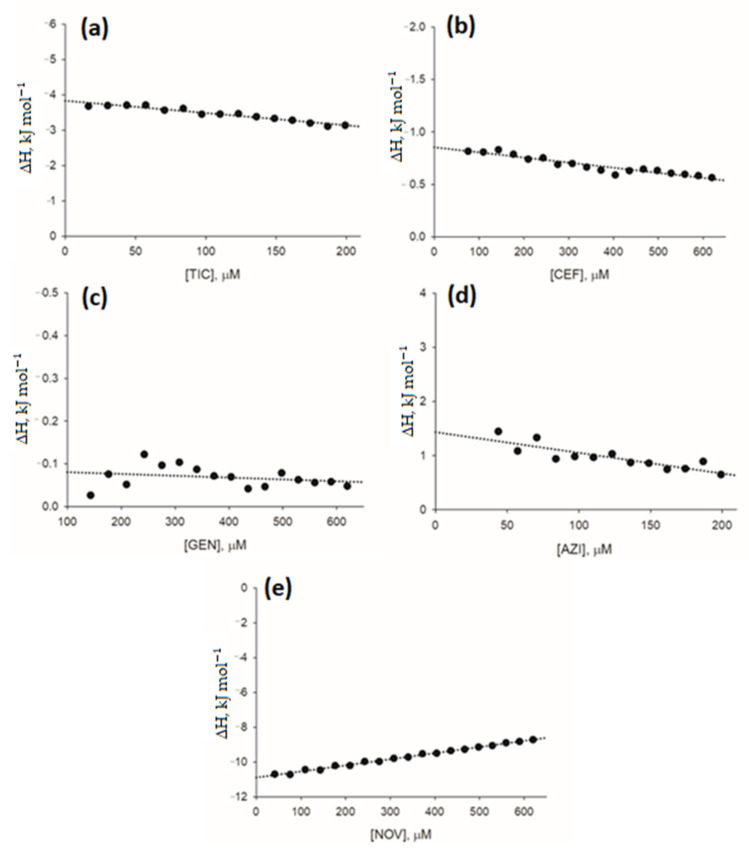
Thermal effects of the antibiotic-caffeine complex formation using isothermal titration calorimetry. Determination of enthalpy change (ΔH, kJ mol^−1^) values for antibiotic-caffeine interactions. Net heat of antibiotic-xanthine interactions after each titration step per mole of titrant added (corrected for heats of buffer-to-caffeine and antibiotic-to-buffer titrations; see Figure S7 for thermograms) are shown as points. (**a**) ticarcillin (TIC)-caffeine interaction; (**b**) cefepime (CEF)-caffene interaction; (**c**) gentamycin (GEN)-caffeine interaction; (**d**) azithromycin (AZI)-caffeine interaction; and (**e**) novobiocin (NOV)-caffeine interaction.

As a result of the limitations of UV-Vis spectroscopy in the quantitative analysis of antibiotics (i.e., ticarcillin, azithromycin, cefepime, and gentamycin) which UV-Vis spectra completely overlapped with the spectrum of caffeine, a complementary method of quantitative analysis of ligand interactions was applied to assess direct interactions between antibiotics and caffeine. ITC measurements also allowed for further characterization of the interactions between novobiocin and caffeine. The analysis of ITC measurements included the calculation of net heat effects of antibiotic-caffeine interactions based on heat effects of antibiotic-caffeine titrations corrected for the heat associated with dilution of the antibiotics and caffeine (as measured in the control buffer titrations). The enthalpy change (ΔH) in antibiotic-caffeine interaction was calculated by extrapolating the net heat effects of interactions towards antibiotic concentration tending to 0. For results of ITC measurements, refer to Figure 4. ΔH values are provided in Table 4.

## 3. Discussion

In this study, we provided evidence for the in vitro potential of caffeine to modulate an activity of a broad range of antibiotics representing diverse classes with distinct antimicrobial mechanisms. Based on the results of the screening phase using disc diffusion assay, we determined the most promising antibiotic-caffeine combinations for which modulatory effects of caffeine were further examined toward a panel of highly relevant human bacterial pathogens. We showed that caffeine enhances the antibacterial activity of five preselected antibiotics: ticarcillin, cefepime, gentamycin, azithromycin, and novobiocin toward at least two bacterial species. Additionally, for most antibiotics tested (except for ticarcillin) we reported synergistic interactions of caffeine for at least one pathogen. Although synergistic effects were not observed for all caffeine-antibiotic combinations and pathogens, in general, relatively low concentrations of caffeine (≥1 mg/mL) substantially reduced the MIC of preselected antibiotics.

Caffeine is one of the most commonly consumed alkaloids worldwide, with well-known pharmacological properties and a well-known safety profile. It is recognized as a safe compound when its daily intake does not exceed 400 mg. The antibacterial activity of caffeine itself is relatively low. The bacteriostatic activity of caffeine at concentrations equivalent to its levels in food (i.e., 0.5% *w*/*v*) was reported towards human pathogen *E. coli* O157 [31]. A study using another *E. coli* strain (*E. coli* K12) showed antibacterial effects of caffeine at 4 mg/mL concentrations [32]. Caffeine at a concentration of 2 mg/mL was also proven effective against the caries-related pathogen *Streptococcus mutans* [33]. These observations are in line with our recent findings on the antibacterial profile of caffeine against ESKAPE pathogens, with MIC values of 4 mg/mL or higher [34]. In this study, antibacterial effects of caffeine alone against a panel of clinical isolates of *S. aureus* with a diverse profile of antibiotic resistance were observed at caffeine concentrations of up to 16 mg/mL for most isolates. The antibacterial activity of caffeine alone observed in this study toward clinical isolates of *S. aureus* was relatively weak, with median MIC values of 16 mg/mL for both MRSA and MSSA strains and slightly larger susceptibility to caffeine of MRSA than MSSA isolates. However, among a handful of isolates with the highest susceptibility to caffeine, two MRSA strains were characterized by a very high level of resistance to a broad range of antibiotics, including resistance to rifamycins, quinolones, fusidic acid, tetracyclines and aminoglycosides (kanamycin, tobramycin, and gentamycin). This might suggest the potential of caffeine to combat *S. aureus* infections difficult to treat with commonly used antibiotics. Still, although the above-mentioned studies confirmed the intrinsic antibacterial effects of caffeine alone, in general, caffeine concentrations required to exert these effects were too high to warrant its efficient usage as an antimicrobial agent in clinical practice.

Therefore, in this study, our main focus was to evaluate the potential of caffeine to act synergistically with commonly used antibiotics. In disc diffusion assays using a panel of 30 structurally diverse antibiotics, we observed both inhibition and potentiation of antimicrobial effects by caffeine, depending on the antibiotic applied. Caffeine exerted relevant inhibitory effects on fluoroquinolones, chloramphenicol, and clindamycin for MRSA as well as MSSA strains. For fluoroquinolones, the observed antagonistic effects of caffeine are consistent with previous studies on ciprofloxacin [32,34], showing that caffeine inhibits the antibacterial potential of this antibiotic. Our study provides evidence that this effect of caffeine is not limited to ciprofloxacin, but rather specific for the whole fluoroquinolone class, at least for gram-positive *S. aureus* strains. The antibacterial activity of fluoroquinolones is based on their inhibitory effects on bacterial gyrase and topoisomerase IV [35]. Thus, the observed inhibitory effects of caffeine can be attributed to its potential to modulate the biological activity of compounds that are capable of direct covalent [36] or non-covalent [37] interactions with DNA and/or which exert their effects through DNA-related enzymes involved in DNA synthesis or maintenance (such as anticancer drugs doxorubicin and mitoxantrone targeting human topoisomerase II [38,39]). Similarly, the inhibitory effects of caffeine on aromatic chloramphenicol, observed in this study, might be explained by the potential of caffeine to sequester aromatic compounds in noncovalent stacking complexes, thus reducing their bioavailability [36,40,41].

In contrast to our observations for fluoroquinolones, in our study, caffeine potentiated rather than inhibited the activity of novobiocin, a potent inhibitor of bacterial gyrase [42]. Caffeine was previously shown to trigger the de-intercalation of aromatic ligands from DNA by forming complexes and reducing the concentration of their free form outside of the DNA (i.e., the interceptor model of de-intercalation) [37]. It may therefore be speculated that these diverse effects of caffeine against antibiotics targeting bacterial gyrase can be dependent on the ability of the antibiotic to directly bind to DNA or to form enzyme-DNA-antibiotic complex, which was proven for fluoroquinolones, but not for aminocoumarins [43,44].

The disc diffusion assay performed for MSSA and MRSA strains showed caffeine-induced potentiation of antibacterial activity of gentamycin, azithromycin, cefepime, and novobiocin. These observations were further confirmed using the checkerboard assay not only for *S. aureus*, but also for gram-negative human pathogens *K. pneumoniae*, *A. baumannii*, and *P. aeruginosa*. At subinhibitory doses, caffeine decreased MIC values of ticarcillin, gentamycin, and cefepime by 16-fold for *S. aureus* MRSA strain, as well as the MIC of azithromycin for *K. pneumoniae*, whereas MICs of cefepime and azithromycin for *P. aeruginosa* and *A. baumannii* were reduced 8-fold. The MIC values of most antibiotics were halved by caffeine at its concentrations of 2 mg/mL or lower for most gram-negative pathogens, whereas MICs of cefepime for *P. aeruginosa* and *A. baumannii* and the MIC of azithromycin for *K. pneumoniae* were halved in the presence of 0.25–0.5 mg/mL of caffeine. Although these concentrations are markedly higher than average peak plasma concentrations of caffeine associated with its intake as a diet constituent [15], strategies such as structural modifications of caffeine toward increasing its adjuvant effects and/or lowering its physiological properties or its local (e.g., topical) and not systemic usage may find direct clinical applications in the era of multidrug resistant microorganisms. Interestingly, the strongest synergistic or additive effects of caffeine on the antibiotics were observed for gram-negative pathogens belonging to the most concerning human pathogens with high prevalence of widely spread multidrug-resistant isolates, in particular within healthcare units, i.e., *A. baumannii* and *P. aeruginosa* [45,46], and for which demand for new therapeutic options is still increasing.

Caffeine as an aromatic compound is capable of forming homo- and hetero-complexes with other aromatic ligands, including antibiotics [30,34,36,38,40,41]. Analysis of direct interactions of caffeine with selected antibiotics in this study revealed complex formation between caffeine and aromatic novobiocin. However, relatively low values of association constant of interaction (K_AC_) and enthalpy change (ΔH) suggest the presence of only weak interactions. For azithromycin and gentamycin, which lack aromatic moiety in their structure, and for cefepime and ticarcillin, which possess only a single aromatic heterocyclic ring, ΔH values were close to 0, indicating no hetero-complexation with caffeine. These findings, along with the previous report on the limited extent of hetero-complexation of caffeine with antibiotics [34] highlight that, in contrast to other biologically active aromatic ligands, for most antibiotics, the sequestration in hetero-complexes with caffeine or other xanthines (such as pentoxifylline), is not a primary factor contributing to observed modulatory effects of xanthines. Fluoroquinolones, the antibiotic class that is capable of DNA binding, can be mentioned as an exception. Following the evidence for direct non-covalent interactions between caffeine and ciprofloxacin [34], hetero-complexation can at least in part explain unexpected inhibitory effects of caffeine toward ciprofloxacin and other fluoroquinolones shown consistently here and in other studies [32,34].

## 4. Materials and Methods

### 4.1. Materials

**Figure 5:**
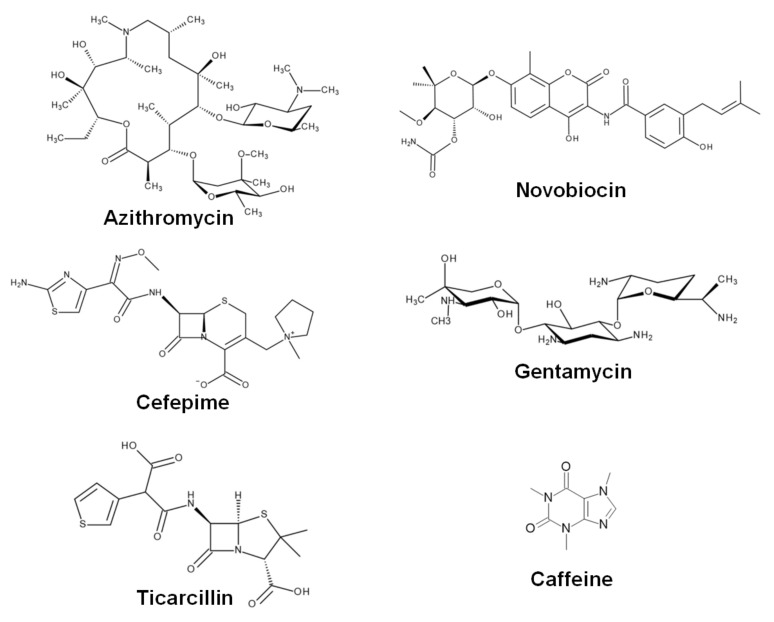
Chemical structures of investigated compounds.

Caffeine (1,3,7-trimethylxanthine) and antibiotics novobiocin sodium salt, cefepime hydrochloride, gentamycin sulfate salt hydrate, azithromycin dihydrate, and ticarcillin disodium salt, were purchased from Sigma-Aldrich (St. Louis, MO, USA). Refer to Figure 5 for the chemical structures of the compounds. For UV-Vis spectroscopy and isothermal titration calorimetry (ITC) measurements, 100 mM sodium phosphate buffer (pH 6.8) was used. The buffer was filtered through a 0.2 μm pore Millex Millipore filter and degassed before experiments. Caffeine stock solutions were prepared in the buffer or deionized water at concentrations of approximately 10^−1^ M, and stored at 4 °C in darkness. Antibiotic stock solutions were prepared by dissolving their weight amounts in the buffer or deionized water immediately before the experiments.

Solid media for bacterial cultivation and testing included tryptone soya agar (TSA, Oxoid Ltd., Basingstone, UK) and Mueller-Hinton agar (BioMaxima, Lublin, Poland). Cation-adjusted Mueller-Hinton broth (CA-MHB) for antimicrobial susceptibility testing using the broth microdilution method was purchased from Beckton Dickinson (BD Difco™ BBL™; Franklin Lakes, NJ, USA).

### 4.2. Antibacterial Screening in a Disc Diffusion Assay

Antibiotic discs were purchased in BioMaxima (Lublin, Poland). Discs containing the following antibiotics (amount in µg unless stated otherwise) were used: penicillin G (1 unit), carbenicillin (100 µg/mL), ticarcillin (75 µg/mL), ampicillin (10 µg/mL), amoxicillin (10 µg/mL), cephazolin (30 µg/mL), cephalexin (30 µg/mL), cefaclor (30 µg/mL), ceftazidime (10 µg/mL), cefotaxime (30 µg/mL), cefepime (30 µg/mL), cephradine (30 µg/mL), bacitracin (10 units), vancomycin (5 µg/mL), colistin sulphate (10 µg/mL), neomycin (10 µg/mL), kanamycin (30 µg/mL), gentamycin (30 µg/mL), erythromycin (15 µg/mL), azithromycin (15 µg/mL), fusidic acid (10 µg/mL), tetracycline (30 µg/mL), doxycycline (30 µg/mL), tigecycline (15 µg/mL), chloramphenicol (30 µg/mL), clindamycin (2 µg/mL), ciprofloxacin (5 µg/mL), moxifloxacin (15 µg/mL), norfloxacin (10 µg/mL), and novobiocin (30 µg/mL).

Antibacterial testing was performed according to the procedure described by EUCAST [47]. The direct colony suspension method was used to prepare bacterial inocula. From an overnight bacterial culture on a TSA plate, two to five colonies were picked using a sterile loop, suspended in phosphate-buffered saline (Sigma-Aldrich, St. Louis, MO, USA), mixed, and adjusted to obtain the microorganism suspension of 0.5 McFarland. The bacterial suspensions containing 5 × 10^7^ CFU were spread thoroughly using a sterile cotton swab to obtain uniform bacterial lawn on plates containing Mueller–Hinton agar without caffeine (control plates) or supplemented with caffeine (0.2 mg/mL, 0.7 mg/mL, 2 mg/mL). Directly afterwards, antibiotic discs were applied to the surface of the inoculated plates. After incubation for 16 to 20 h at 35 °C, the zones of growth inhibition around the discs were evaluated. Disc diffusion tests were performed as three technical and at least three biological replicates.

### 4.3. Antibacterial Susceptibility Testing Using Broth Microdilution Method

The antimicrobial potential of selected compounds was evaluated using the broth microdilution method. The following bacterial strains were used throughout the study: gram-positive *Staphylococcus aureus* ATCC 25923 and *Staphylococcus aureus* ATCC MRSA 43300; gram-negative *Pseudomonas aeruginosa* ATCC 27853 and *Acinetobacter baumannii* ATCC 19606, and *Klebsiella pneumoniae* ATCC 700603.

Additionally, the antibacterial potential of caffeine was investigated towards a collection of approximately 100 clinical isolates of *Staphylococcus aureus* (including ca. 50 MRSA isolates). They were obtained previously from hospitalised and outpatient clinic patients and collected as part of departmental biobank of microorganisms at the Intercollegiate Faculty of Biotechnology UG&MUG. The bacteria included isolates from the nasal cavity, throat, bronchi, blood, rectum, wounds, ulcerations, skin, ear, marrow cavity, and vascular catheters. All isolates had a well-characterized profile of resistance to antibiotics, including glycopeptides, rifamycins, quinolones, fusidic acid, tetracylines, beta-lactams, aminoglycosides, and macrolides. All MRSA isolates carried modifications within the penicillin-binding protein (mecA) gene, whereas most MSSA isolates produced penicillinase. Almost all isolates were susceptible to glycopeptides, with only one heterogeneously resistant to vancomycin (hetero-VISA). Most isolates were susceptible to rifamycins and fusidic acid. In all isolates, resistance to aminoglycoside kanamycin was reported, and 29 isolates were also resistant to tobramycin and gentamycin. Approximately half of the isolates were partially resistant, and another half susceptible to, tetracyclines. Most MRSA isolates were resistant or partially resistant to quinolones, whereas most MSSA isolates were susceptible to quinolones. For the detailed characteristics of the isolates used, refer to Table S1.

The procedure applied was according to the CLSI guidelines [48]. Sterile, polystyrene, flat-bottom 96-well plates for non-adherent cultures (NEST^®^) were used. Minimal inhibitory concentration (MIC) was defined as the lowest concentration of an investigated compound with no visible bacterial growth observed after a 24 h stationary incubation at 37 °C. The following concentration gradients (obtained by serial two-fold dilutions of the test medium) were applied: for antibiotics, from 128 to 0.015625 µg/mL; for caffeine, from 16 to 1 mg/mL. From the prepared solutions, 100 µL aliquots were transferred into the 96-well plates. Next, each well was inoculated with a 10 µL aliquot of a bacterial suspension containing approximately 1 × 10^5^ CFU/mL. Bacterial suspensions were obtained from liquid cultures in CA-MHB (6 h, 37 °C, 150 rpm), diluted with a fresh medium. The same procedure was applied to evaluate the antibacterial potential of caffeine against clinical isolates of *S. aureus* (MRSA and MSSA). To analyze the interactions of antibiotics and caffeine, a checkerboard titration method was used. This was accomplished by applying a two-dimensional (antibiotic-caffeine) combination of concentration gradients. For agents with antibacterial activity (i.e., when MIC value was determined), the following gradient was applied: 2 × MIC, 1 × MIC, 0.5 × MIC, 0.25 × MIC, 0.125 × MIC, 0.06 × MIC, and 0.03 × MIC. Antibiotics without antibacterial potential were applied at concentration gradient: 4096, 2048, 1024, 512, 256, 128, and 64 µg/mL. For caffeine-resistant strains, caffeine was applied starting from the highest tested concentration, i.e., 16 mg/mL, and the following concentrations were tested: 16, 8, 4, 2, 1, 0.5 and 0.25 mg/mL. Two methods were used to evaluate the results of checkerboard experiments: calculation of the Fractional Inhibitory Concentration Index (FICI) for each tested combination (according to Odds) [29], and isobologram analysis [49]. The FICI was applied for the combination of the lowest concentration of both compounds inhibiting bacterial growth, and was calculated according to the following equation:

FICI = (MIC_A+B_/MIC_A_) + (MIC_B+A_/MIC_B_), (1)

where

MIC_A+B_—the lowest concentration of compound A in the presence of the lowest concentration of compound B at which inhibitory effect is observed,
MIC_A_—MIC of compound A tested alone,
MIC_B+A_—the lowest concentration of compound B in the presence of the lowest concentration of compound A at which inhibitory effect is observed,
MIC_B_—MIC of compound B tested alone.

If no antimicrobial effect on particular bacterial pathogens (MIC > 16 mg/mL) was determined for caffeine, its highest used concentration, i.e., 16 mg/mL, was considered equal to or lower than 0.5 × MIC. The following types of interaction were defined: (i) synergistic for FICI ≤ 0.5, (ii) additive for FICI between 0.5 and 2.0, and (iii) antagonistic for FICI ≥ 4.0 [29]. Three biological replicates of all experiments were performed with at least 24 h intervals.

### 4.4. UV-Vis Spectroscopy Measurements and Calculations

For UV-Vis spectroscopy measurements, quartz cuvettes (1 cm light path) were filled with antibiotics dissolved in a 0.1 M sodium phosphate buffer (pH 6.8). The aliquots were titrated with 5–150 μL of caffeine stock solution. The absorption spectra of antibiotics alone and antibiotic-caffeine mixtures were measured using a Jena Analytic Specord 50 Plus spectrophotometer equipped with a Peltier thermostat (25 ± 0.1 °C) at 0.5 nm intervals, and stored in a digital form. To maintain stable measurement conditions, following each titration and prior to the measurement, the solution was gently mixed and the cuvette was placed in a thermostatted holder for approximately 5 min. Absorption spectra were expressed in a form of molar absorption coefficient (ε_λ_, M^−1^ cm^−1^).

To observe only changes in the optical properties of antibiotics, the UV-Vis spectra were analyzed in the range of wavelengths above 320 nm, for which light absorption of caffeine is negligible (see Figure S6). To calculate the spectrum of the antibiotic complexed with caffeine, molar extinction coefficients (for each wavelength) were extrapolated to the concentration ratio of antibiotic to caffeine tending to 0. To estimate the concentration of antibiotic in its free form and in hetero-complex with caffeine, the spectra of antibiotic-caffeine mixtures were decomposed into a weighted sum of components (antibiotic in its free form and antibiotic hetero-complexed with caffeine) by non-linear regression analysis.

Mixed association constant values (K_AC_) for antibiotic-caffeine complexation were calculated using a statistical-thermodynamical model of mixed aggregation described by Zdunek et al. [30]. The model describes interactions in 2-component ligand-caffeine mixtures, with one component, C (caffeine) capable of both homo- and hetero-complexation, and the other component, A (antibiotic), capable of only hetero-complexation with caffeine. The constant value of caffeine homo-complexation reported previously [50] was deployed into the model. The calculations were performed using SigmaPlot 11 (Systat Software, Inc., San Jose, CA, USA), Microsoft Office Excel (Microsoft, Redmond, WA, USA), and Mathcad Prime 6 (Parametric Technology Corporation, Boston, MA, USA) software.

### 4.5. Isothermal Titration Calorimetry (ITC)

In isothermal titration calorimetry (ITC) experiments, an AutoITC isothermal titration calorimeter (MicroCal, Malvern Panalytical Inc., WR14 1XZ Malvern, UK) was used, with a reference cell (containing deionized water) and a sample cell of 1.4491 mL volume. The cell containing deionized water was used as the reference. All experiments were performed in deionized water at 25 °C after degassing. The experiment consisted of 20 injections (2 μL for the first injection, 10.02 μL for the following injections) of the antibiotic solution (2 mM for azithromycin and ticarcillin, 5 mM for cefepime, novobiocin, and gentamycin) into the sample cell initially containing 15 mM caffeine. Injections (each lasting 20 s) were conducted in 5-min intervals to ensure the return of titration peak to the baseline prior to the next injection. Background titrations were performed using identical titrants with deionized water placed in the sample cell or using deionized water with caffeine solution contained in the sample cell. To account for the heat of dilution, heats corresponding to background titrations were subtracted from each experimental titration. To achieve a homogeneous mixing in the cell, the stirrer speed was established at 300 rpm. Initial 2 μL injections were removed from each data set before analysis to account for the effect of titrant diffusion from the syringe tip to the sample cell during the equilibration process. Origin 7 software (MicroCal) was used to process the data and to calculate the heat normalized per mole of the injectant.

## 5. Conclusions

In conclusion, in this study, we showed that caffeine modulates the antibacterial activity of a broad range of antibiotics commonly used in clinical settings. The most promising synergistic or additive effects were observed for gentamycin, azithromycin, cefepime, and novobiocin applied against gram-negative pathogens. These findings may serve as a basis for further studies to evaluate the relevance of caffeine as a potential adjuvant in antibacterial therapies.

## Figures and Tables

**Table 1:** Caffeine impact on antibacterial activity of antibiotics in a disc diffusion assay.

*Staphylococcus Aureus* Newman				*Staphylococcus aureus* Mu		
	CAF 0.2 mg/mL	CAF 0.7 mg/mL	CAF 2 mg/mL	CAF 0.2 mg/mL	CAF 0.7 mg/mL	CAF 2 mg/mL
**Penicillins**						
Penicillin	0.9 (0.6)	0.4 (1.1)	2.7 (1.4)	0.0 (0.0)	0.8 (1.0)	1.2 (1.0)
Carbenicillin	1.3 (0.6)	2.3 (1.2)	3.7 (0.6)	0.0 (0.0)	1.3 (11.5)	0.0 (0.0)
Ticarcillin	3.8 (1.9)	4.8 (1.3)	5.5 (0.6)	0.0 (0.0)	0.0 (0.0)	0.0 (0.0)
Ampicillin	−4.0 (1.4)	−1.0 (1.4)	−0.5 (0.7)	2.0 (0.0)	1.0 (0.0)	0.5 (0.7)
Amoxicillin	0.8 (1.5)	1.3 (1.9)	0.3 (1.3)	−0.3 (1.0)	−0.5 (0.6)	0.0 (0.8)
**Cephalosporins**						
Cephazolin	−1.8 (1.3)	0.3 (1.0)	−0.3 (2.1)	0.3 (1.2)	2.3 (0.6)	2.0 (1.4)
Cephalexin	1.7 (1.5)	1.0 (1.0)	0.3 (1.5)	0.0 (0.0)	0.3 (0.6)	0.0 (0.0)
Cefaclor	0.7 (0.6)	1.3 (1.5)	0.0 (0.0)	0.7 (2.1)	0.3 (1.5)	0.0 (0.0)
Ceftazidime	−0.3 (1.2)	1.0 (1.7)	0.0 (3.0)	0.0 (0.0)	0.0 (0.0)	0.0 (0.0)
Cefotaxime	0.7 (1.2)	4.3 (1.5)	7.0 (2.0)	0.0 (1.0)	3.0 (3.0)	−0.7 (11.0)
Cefepime	3.7 (0.6)	5.0 (1.0)	5.3 (0.6)	−0.3 (0.6)	2.7 (0.6)	5.0 (1.0)
Cephradine	−4.0 (1.0)	−4.3 (1.2)	−7.7 (1.2)	0.0 (0.0)	0.0 (0.0)	0.0 (0.0)
**Nonribosomal peptide antibiotics**						
Bacitracin	0.3 (1.2)	−0.3 (0.6)	−3.0 (0.0)	1.7 (0.6)	4.3 (0.6)	3.0 (1.0)
Vancomycin	2.0 (1.7)	2.3 (0.6)	2.0 (1.7)	1.3 (0.6)	2.7 (1.2)	2.0 (1.7)
Colistin sulphate	2.3 (0.6)	4.0 (0.0)	4.0 (1.0)	0.3 (0.6)	0.3 (1.5)	0.0 (1.7)
**Aminoglycosides**						
Neomycin	3.7 (1.5)	2.3 (0.6)	3.7 (0.6)	0.0 (0.0)	0.0 (0.0)	0.0 (0.0)
Kanamycin	0.5 (0.6)	1.0 (1.2)	0.8 (1.3)	10.5 (7.9)	2.5 (5.0)	2.5 (5.0)
Gentamycin	0.0 (1.0)	1.0 (1.0)	3.7 (1.2)	3.3 (5.8)	10.3 (0.6)	11.3 (1.2)
**Macrolides**						
Erythromycin	0.4 (1.6)	0.0 (2.6)	2.8 (1.1)	0.2 (0.5)	0.1 (0.3)	2.7 (4.9)
Azithromycin	2.3 (1.5)	2.7 (1.2)	3.0 (1.7)	9.7 (9.1)	10.0 (9.2)	0.0 (0.0)
**Fusidic acid**	0.5 (0.7)	1.0 (0.0)	2.0 (0.0)	−3.5 (0.7)	−0.5 (0.7)	3.5 (2.1)
**Tetracyclines**						
Tetracycline	−1.0 (2.0)	−0.3 (1.5)	1.0 (1.0)	0.0 (1.7)	−0.7 (0.6)	0.3 (1.2)
Doxycycline	1.3 (0.6)	3.0 (1.0)	4.3 (0.6)	0.7 (0.6)	1.3 (1.2)	−1.3 (2.1)
Tigecycline	−2.0 (1.0)	−1.0 (0.0)	−2.0 (0.0)	2.0 (1.7)	2.0 (0.0)	3.0 (1.0)
Chloramphenicol	−3.4 (1.1)	−1.0 (2.1)	1.4 (0.9)	−3.3 (1.3)	−5.3 (1.0)	−6.5 (1.3)
**Lincosamides**						
Clindamycin	−3.0 (1.0)	−3.0 (2.0)	0.0 (3.6)	0.0 (0.0)	0.5 (0.7)	−1.0 (0.0)
**Fluoroquinolones**						
Moxifloxacin	0.7 (1.2)	0.7 (1.2)	0.3 (2.3)	1.0 (2.0)	−1.0 (2.6)	−1.7 (3.8)
Norfloxacin	−1.0 (0.0)	−2.3 (0.6)	−5.0 (1.0)	0.7 (0.6)	0.3 (2.5)	−1.3 (0.6)
Ciprofloxacin	−3.3 (2.5)	−3.0 (3.0)	−2.3 (2.5)	0.3 (2.1)	−3.3 (1.5)	−3.7 (1.2)
**Aminocoumarins**						
Novobiocin	1.8 (1.0)	3.8 (1.5)	5.8 (1.0)	2.3 (0.5)	4.3 (2.1)	4.0 (5.4)

CAF, caffeine. Values correspond to differences (in mm) in growth inhibition diameter observed on corresponding plates with and without caffeine (negative values mean a reduction in inhibition zone; positive values mean an increase in inhibition zone). Mean (standard deviation) values of at least three biological replicates are shown. Concentrations of caffeine in an agar growth medium are given.

**Table 2:** The impact of caffeine on the antibacterial activity of selected antibiotics toward a panel of human pathogens.

	MIC_A_ [µg/mL]	MIC_A+CAF_ [µg/mL]	FICI	Type of Interaction
***Staphylococcus aureus*** **ATCC 25923**				
Ticarcillin	2	1	≥0.75	Additive
Cefepime	4	4	≥1.007	No interaction
Gentamycin	0.5	0.5	≥1.007	No interaction
Azithromycin	2	0.5	≥0.75	Additive
Novobiocin	0.5	1	≥1.007	Antagonistic
***Staphylococcus aureus*** **ATCC MRSA 43300**				
Ticarcillin	256	16	≥2.007	Dose-dependent (antagonistic/additive)
Cefepime	64	4	≥1.007	Antagonistic
Gentamycin	2	0.125	≤0.28	Synergistic
Azithromycin	>512	Not evaluable		
Novobiocin	0.25	1	≥2.007	Antagonistic
***Pseudomonas aeruginosa*** **ATCC 27853**				
Ticarcillin	32	16	≥0.75	Dose-dependent (antagonistic/additive)
Cefepime	8	1	≥0.375	Synergistic
Gentamycin	4	1	≥0.5	Synergistic/additive
Azithromycin	128	16	≥0.5	Synergistic/additive
Novobiocin	512	128	≥0.625	Additive
***Klebsiella pneumoniae*** **ATCC 700603**				
Ticarcillin	>4096	Not evaluable		
Cefepime	4	8	1.007	Antagonistic
Gentamycin	16	8	0.75	Additive
Azithromycin	64	4	0.375	Synergistic
Novobiocin	4096	1024	0.5	Synergistic
***Acinetobacter baumannii*** **ATCC 19606**				
Ticarcillin	16	8	1	Additive
Cefepime	64	8	0.5	Synergistic
Gentamycin	128	64	1.5	Antagonistic
Azithromycin	64	8	0.75	Additive
Novobiocin	16	4	0.75	Additive

MIC_A_, minimal inhibitory concentration of an antibiotic; MIC_A+CAF_, minimal inhibitory concentration of an antibiotic tested with caffeine at the highest sub-inhibitory caffeine concentration (corresponding to MIC/2 or, when MIC > 16 mg/mL, the highest tested concentration); and FICI, fractional inhibitory concentration index, calculated according to Odds [29] for each tested combination of antibiotic and caffeine.

**Table 3:** Antibacterial activity of caffeine against clinical isolates of *Staphylococcus aureus* in a broth microdilution assay.

MIC of Caffeine, mg/mL	MRSA (*N* = 48)		MSSA (*N* = 51)	
	*n*	%	*n*	%
4	2	4	2	4
8	12	25	8	16
16	21	44	28	55
>16	13	27	13	25

*N*, total number of samples; *n*, number of samples in categories.

**Table 4:** Biophysical analysis of non-covalent interactions between caffeine and selected antibiotics.

Antibiotic	K_AC_ (SE) [M^−1^]	ΔH (SE) [kJ·mol^−1^]
Ticarcillin	Not evaluable ^1^	−3.85 (0.04)
Cefepime	Not evaluable ^1^	−0.84 (0.02)
Gentamycin	Not evaluable ^1^	−0.08 (0.02)
Azithromycin	Not evaluable ^1^	1.42 (0.08)
Novobiocin	30.51 (1.72)	−10.89 (0.04)

SE, standard error. ^1^ As a result of UV-Vis spectra overlap, quantitative analysis of antibiotic-caffeine interactions was not possible.

## Data Availability

Data are contained within the article and Supplementary Materials.

## Supplementary Materials

The following supporting information can be downloaded at: https://www.mdpi.com/article/10.3390/ph15070872/s1, Supplementary Figures S1–S5. Impact of caffeine on antibacterial activity of antibiotics in *Staphylococcus aureus* ATCC 25923 (Figure S1), *Staphylococcus aureus* ATCC MRSA 43300 (Figure S2), *Pseudomonas aeruginosa* ATCC 27853 (Figure S3), *Klebsiella pneumoniae* ATCC 700603 (Figure S4), and *Acinetobacter baumannii* ATCC 19606 (Figure S5) strains using microbroth dilution assay and checkerboard methodology. (a) effects on ticarcillin; (b) effects on cefepime; (**c**) effects on gentamycin; (d) effects on azithromycin; and (e) effects on novobiocin. FIC Index, Fractional Inhibitory Concentration Index calculated for each tested combination of antibiotic and caffeine according to Odds [29]. Supplementary Figure S6. Overlap of UV-Vis absorption spectra of caffeine (94.6 µM), cefepime (54.8 µM), gentamycin (18.3 mM), ticarcillin (25.9 µM), and azithromycin (442.7 µM). Supplementary Figure S7. Isothermal titration calorimetry thermograms for analysis of antibiotic-caffeine interactions. (a) ticarcillin (TIC)-caffeine interaction; (b) cefepime (CEF)-caffene interaction; (c) gentamycin (GEN)-caffeine interaction; (d) azithromycin (AZI)-caffeine interaction; and (e) novobiocin (NOV)-caffeine interaction. Titration of caffeine with antibiotic is shown in green water with caffeine—in black and antibiotic with water—in red. Supplementary Table S1. Characteristics and antibacterial effects of caffeine toward clinical isolates of *Staphylococcus aureus*.
