# Supplementary material for: New Life of an Old Drug: Caffeine as a Modulator of Antibacterial Activity of Commonly Used Antibiotics
Source: Pharmaceuticals (Basel). 2022 Jul 15;15(7):872. doi: 10.3390/ph15070872 (PMC9315996; doi:10.3390/ph15070872)
Supplement: Supplementary file 1 [file pharmaceuticals-15-00872-s001.zip › pharmaceuticals-1798279-supplementary.pdf]

Article

New life of an old drug: caffeine as a modulator of antibacterial activity of commonly used antibiotics

Anna Woziwodzka[1,*], Marta Krychowiak-Maśnicka[2], Grzegorz Gołuński[1], Anna Łosiewska[1], Agnieszka Borowik[1,3], Dariusz Wyrzykowski[4] and Jacek Piosik[1]

1. Laboratory of Biophysics, Intercollegiate Faculty of Biotechnology University of Gdansk and Medical University of Gdansk, 80-307 Gdansk, Poland
2. Laboratory of Biologically Active Compounds, Intercollegiate Faculty of Biotechnology University of Gdansk and Medical University of Gdansk, 80-307 Gdansk, Poland
3. Aging and Metabolism Research Program, Oklahoma Medical Research Foundation, Oklahoma City, OK, USA
4. Department of Inorganic Biological Chemistry, Faculty of Chemistry, University of Gdansk, 80-308 Gdansk, Poland

* Correspondence: anna.woziwodzka@ug.edu.pl,; Tel.: +48 58 523 6310

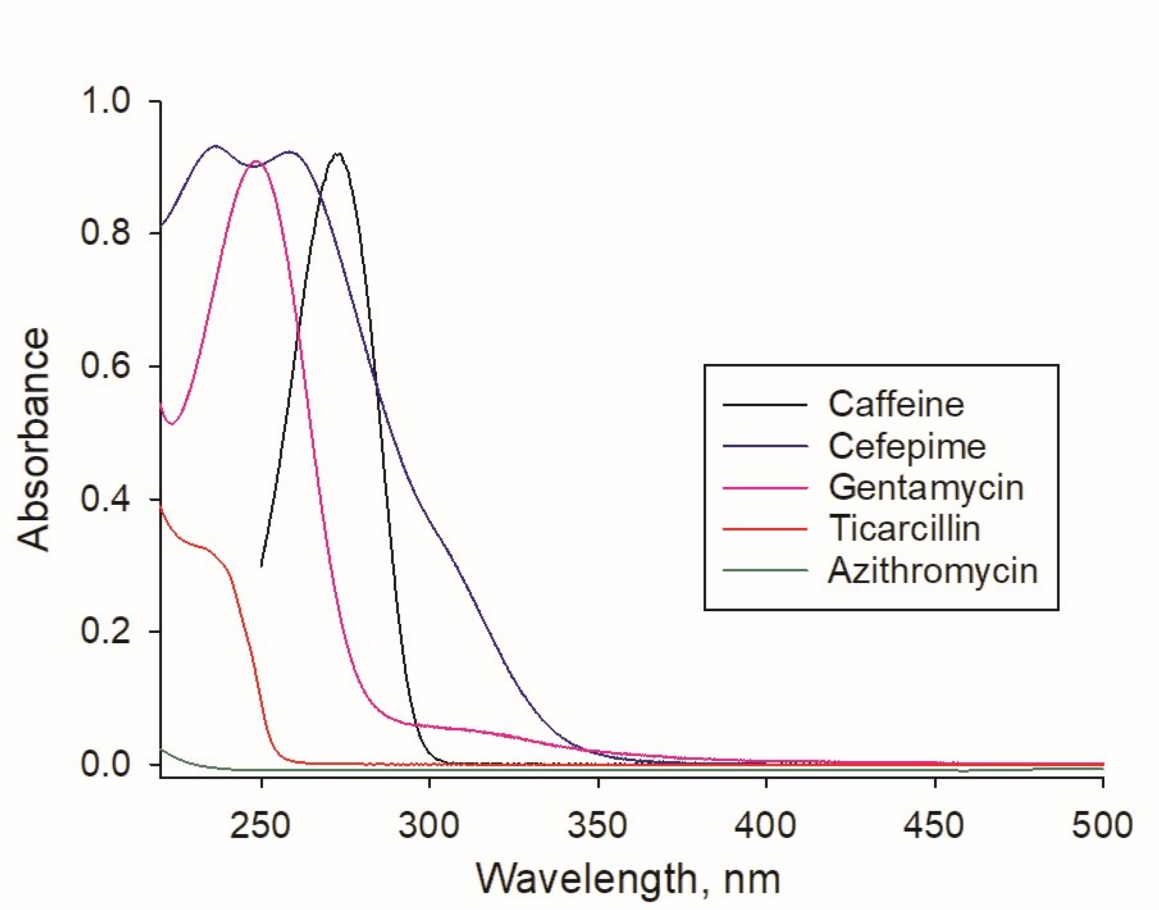

Figure S1. Overlap of UV-Vis absorption spectra of caffeine (94.6 μM), cefepime (54.8 μM), gentamycin (18.3 mM), ticarcillin (25.9 μM), and azithromycin (442.7 μM).

Figure S2a

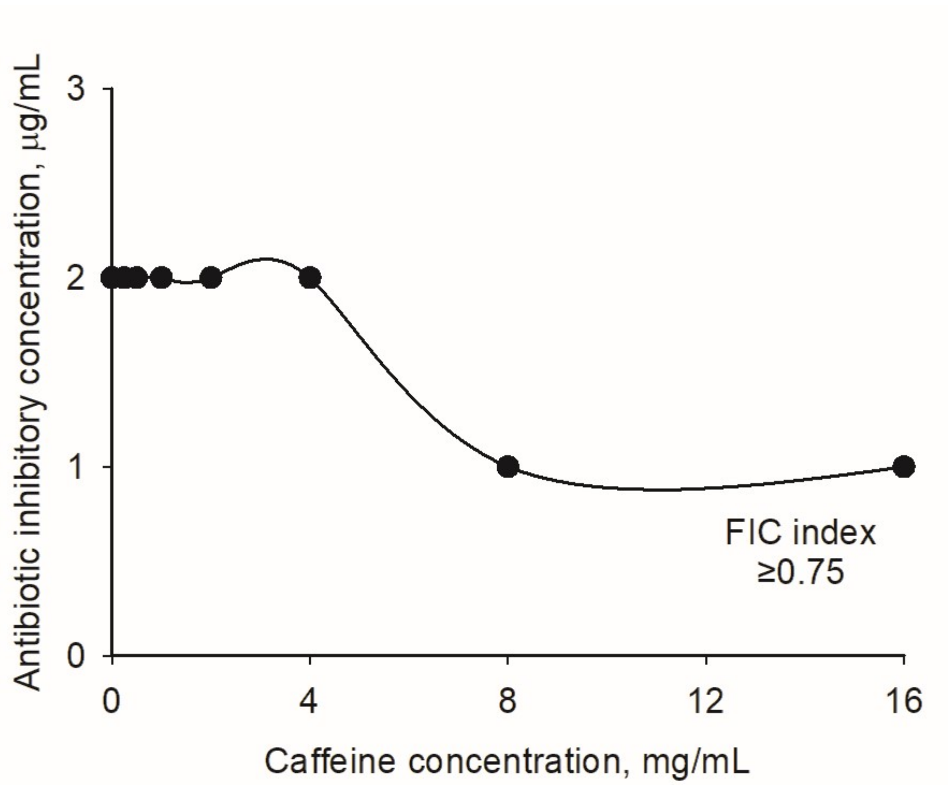

Figure S2b

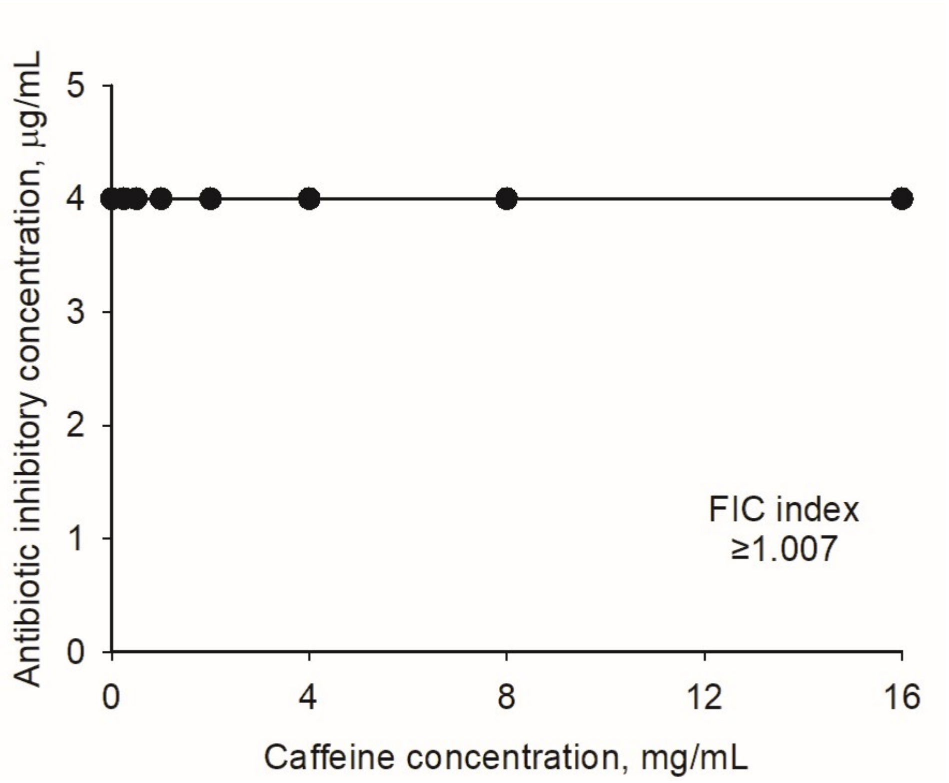

Figure S2c

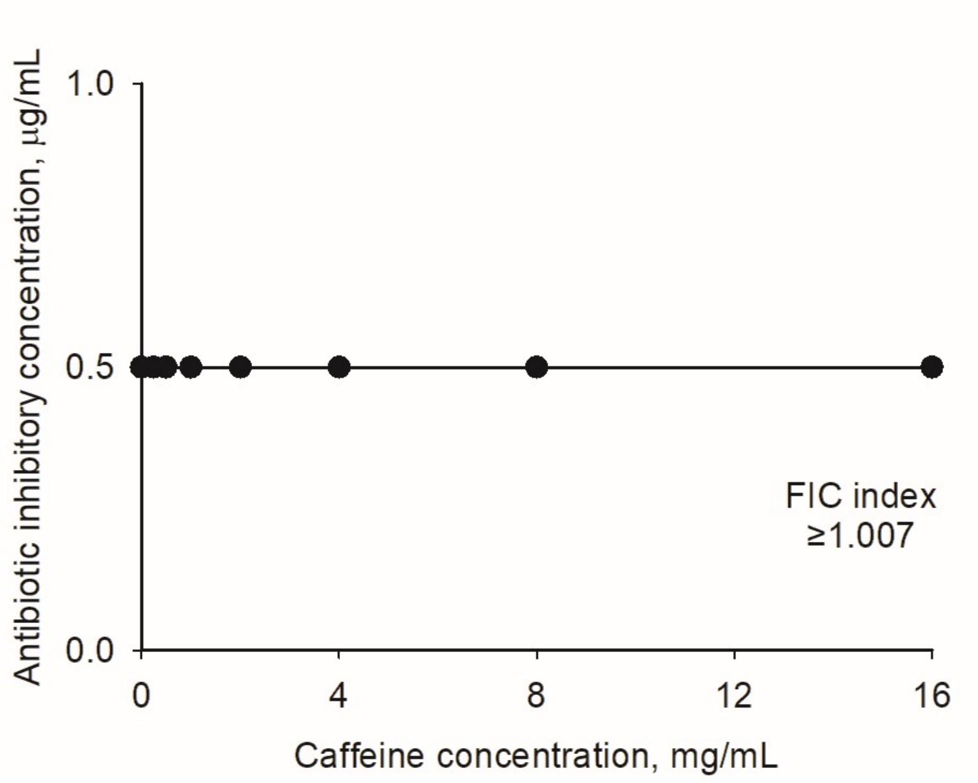

Figure S2d

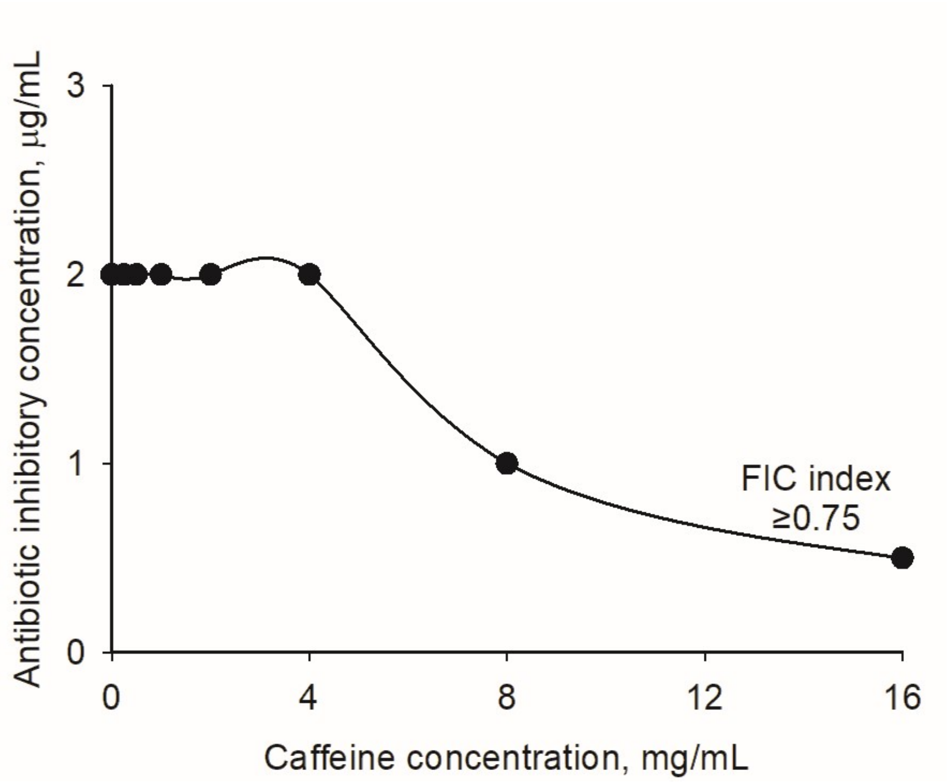

Figure S2e

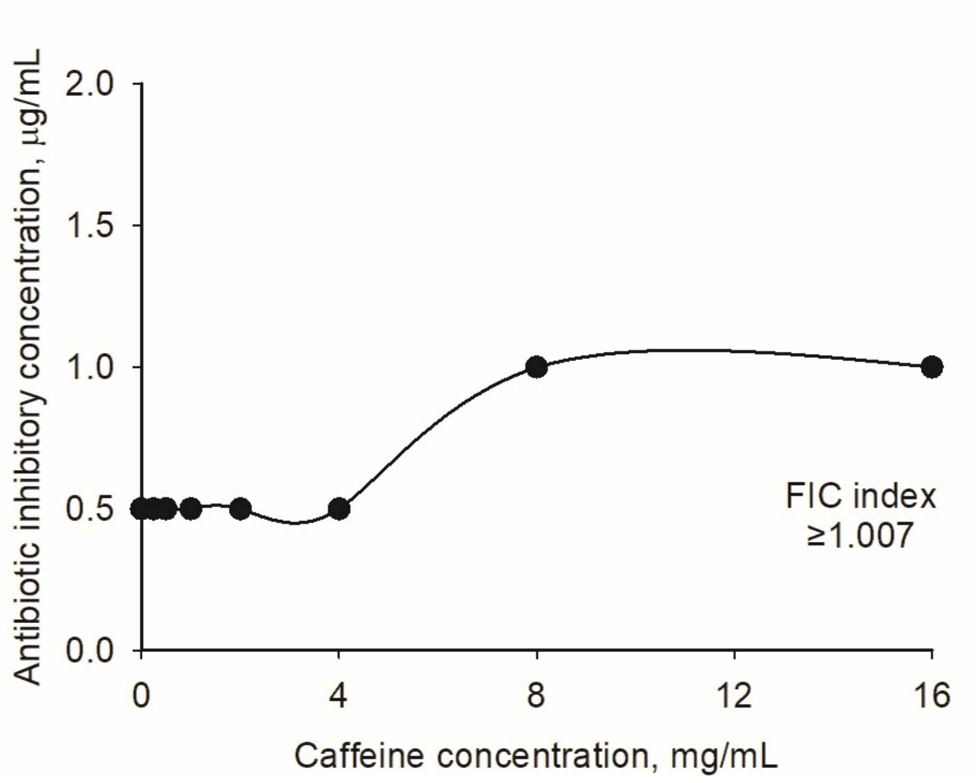

Figure S2. Impact of caffeine on antibacterial activity of antibiotics in *Staphylococcus aureus* ATCC 25923 strain using microbroth dilution assay and checkerboard methodology. (**a**), effects on ticarcillin; (**b**), effects on cefepime; (**c**), effects on gentamycin; (**d**), effects on azithromycin; (**e**), effects on novobiocin. FIC Index, Fractional Inhibitory Concentration Index calculated for each tested combination of antibiotic and caffeine according to Odds [26]

Figure S3a

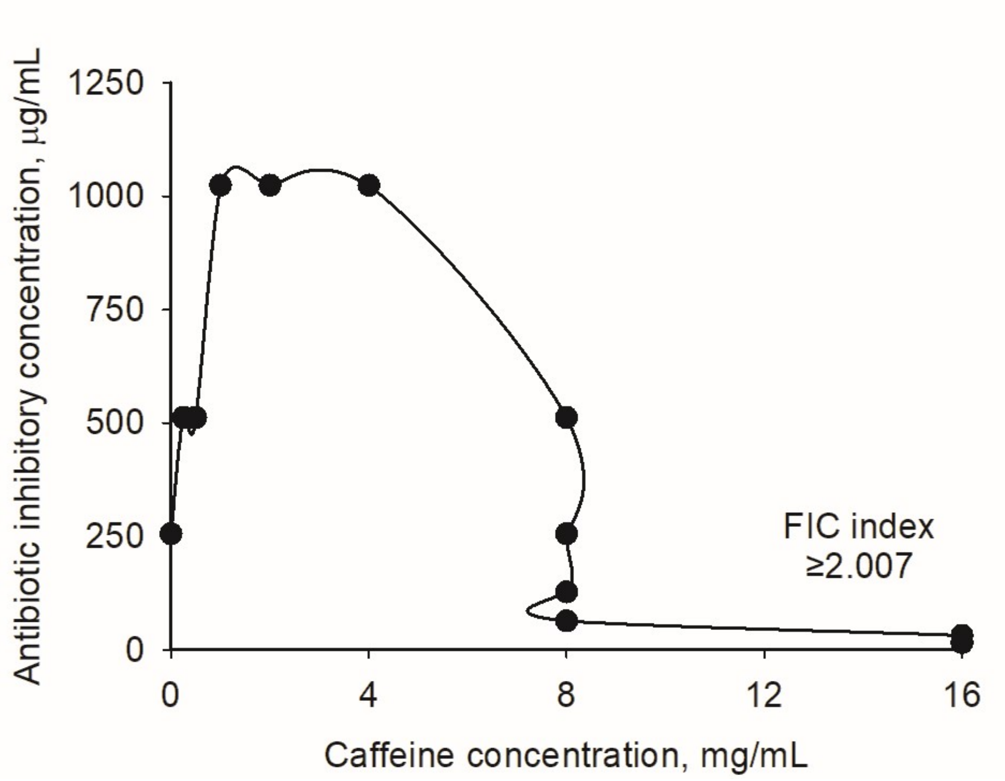

Figure S3b

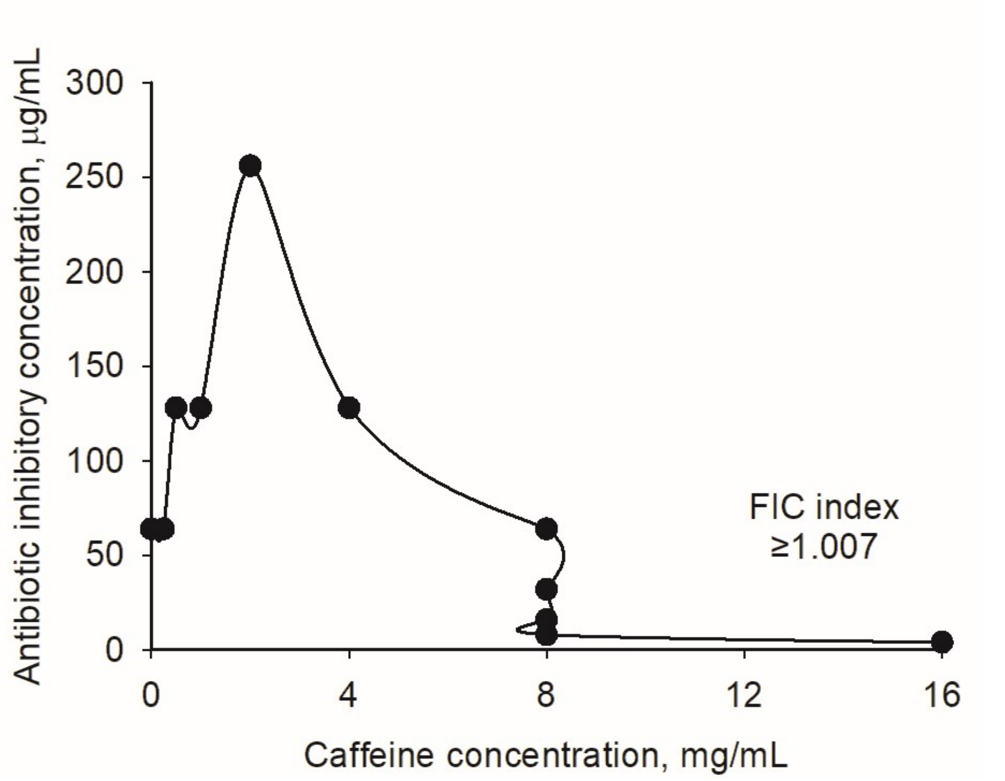

Figure S3c

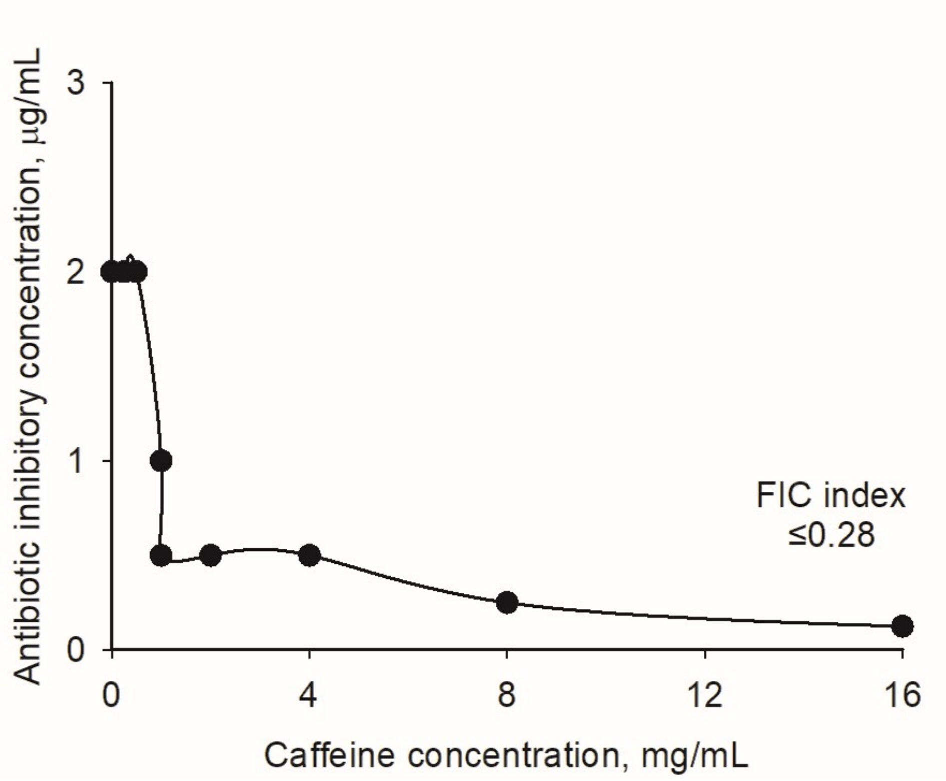

Figure S3d

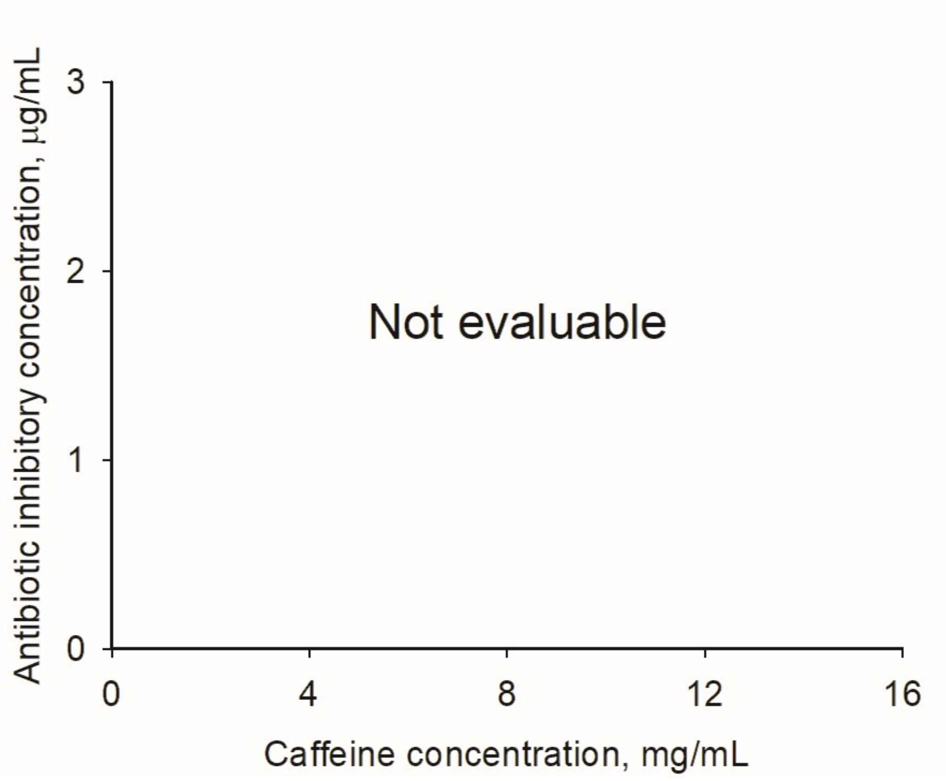

Figure S3e

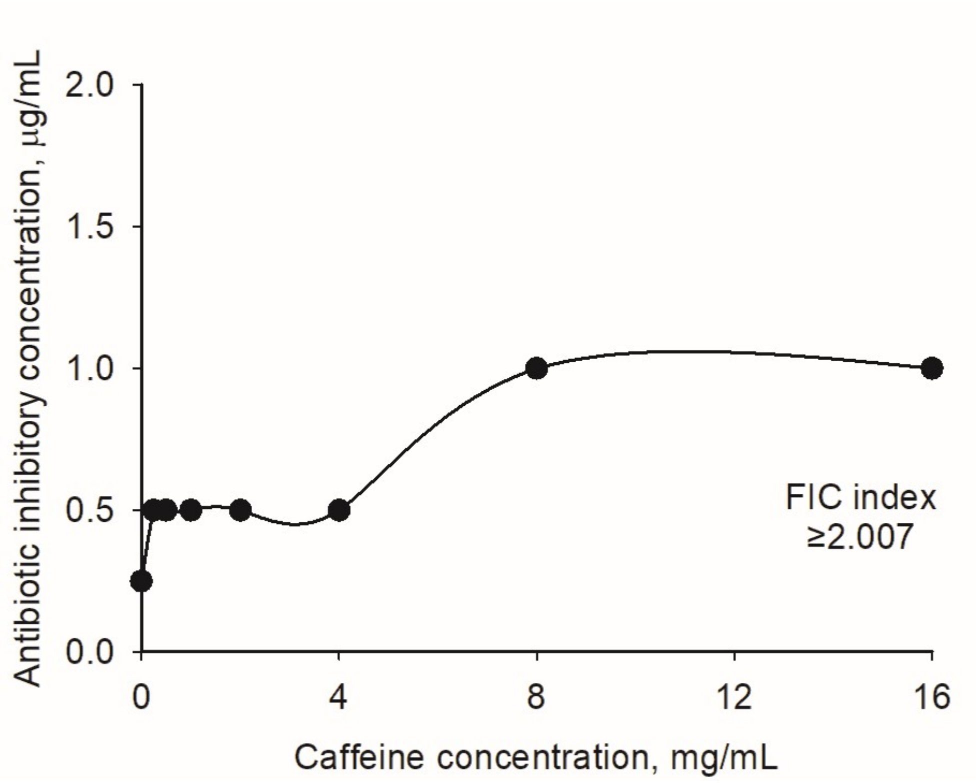

Figure S3. Impact of caffeine on antibacterial activity of antibiotics in *Staphylococcus aureus* ATCC MRSA 43300 strain using microbroth dilution assay and checkerboard methodology. (**a**), effects on ticarcillin; (**b**), effects on cefepime; (**c**), effects on gentamycin; (**d**), effects on azithromycin; (**e**), effects on novobiocin. FIC Index, Fractional Inhibitory Concentration Index calculated for each tested combination of antibiotic and caffeine according to Odds [26]

Figure S4a

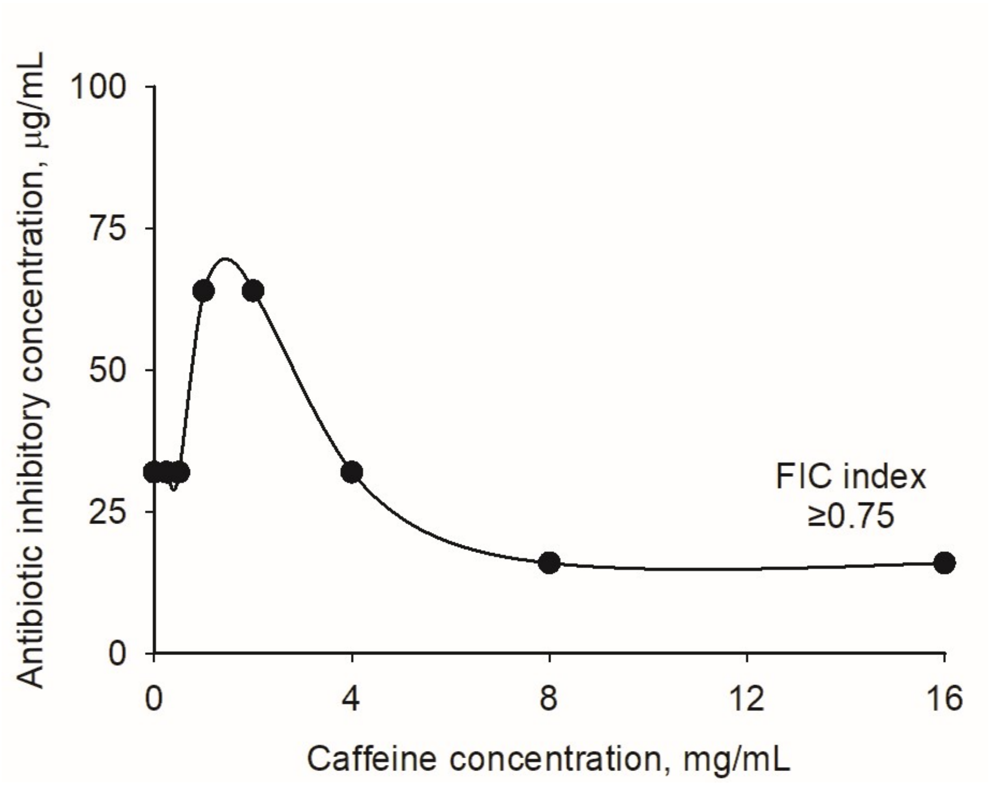

Figure S4b

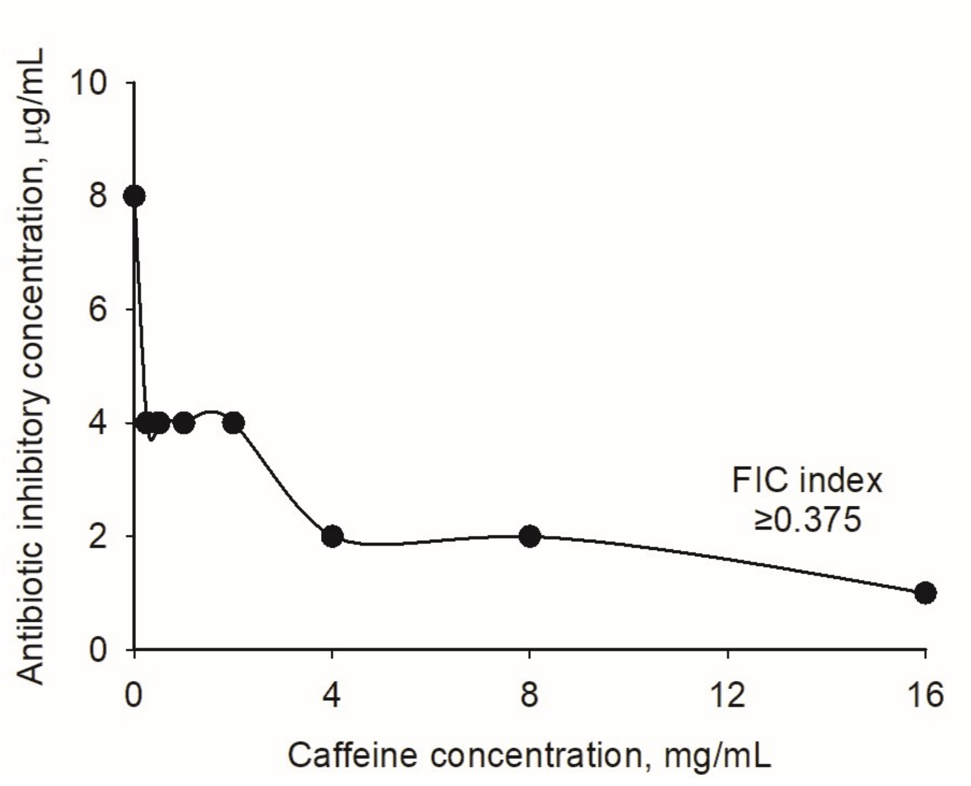

Figure S4c

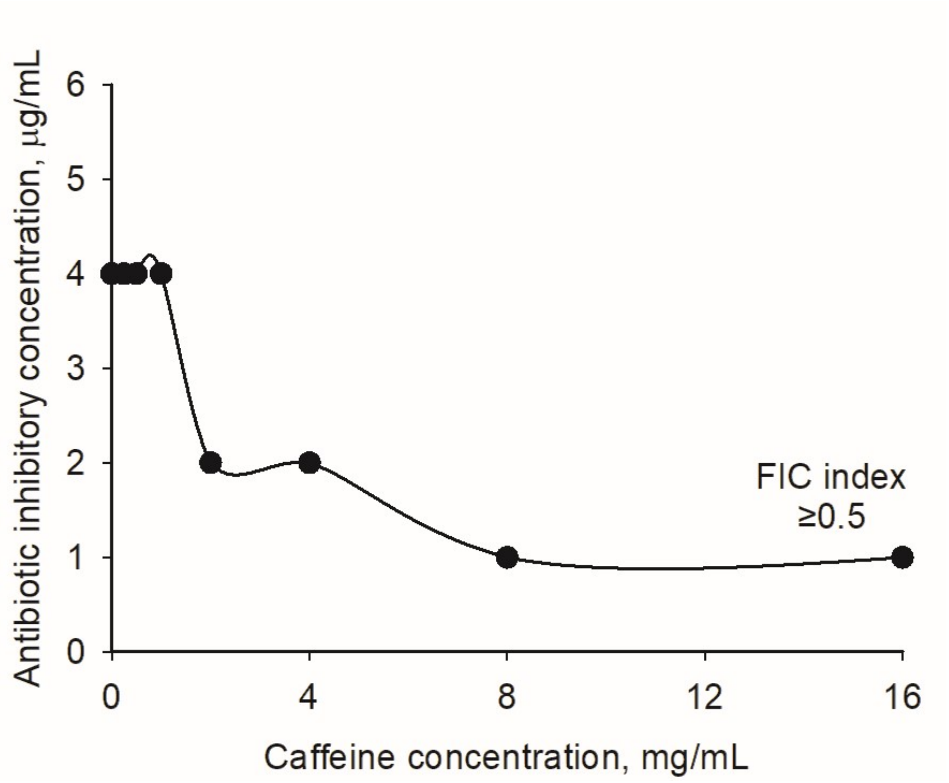

Figure S4d

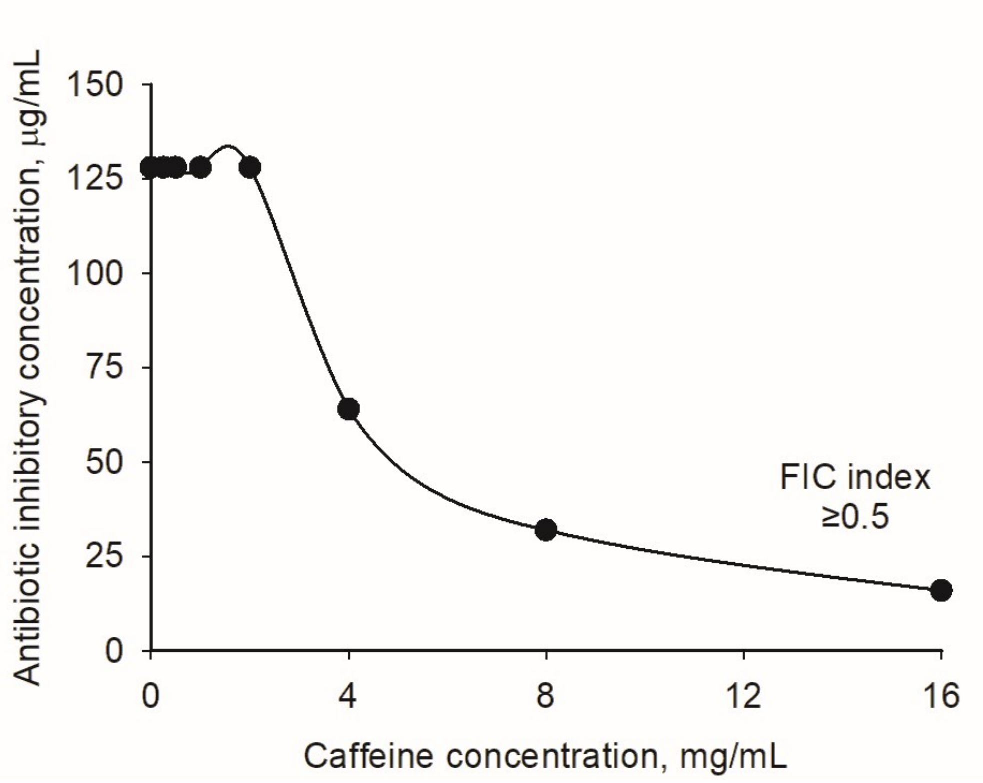

Figure S4e

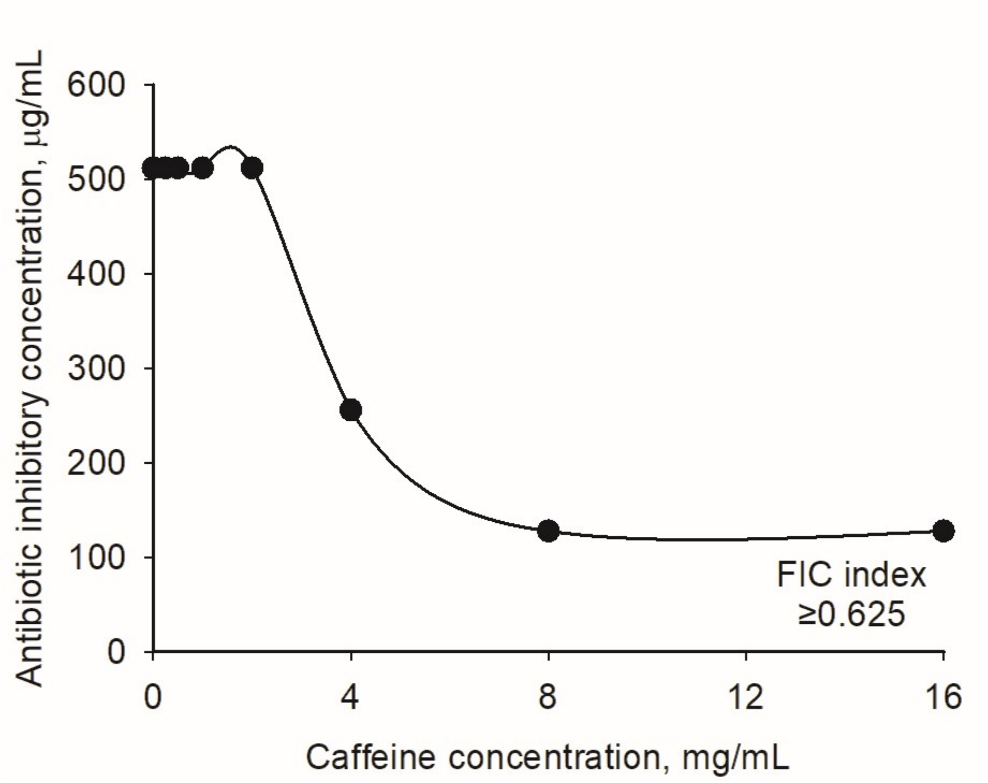

Figure S4. Impact of caffeine on antibacterial activity of antibiotics in *Pseudomonas aeruginosa* ATCC 27853 strain using microbroth dilution assay and checkerboard methodology. (**a**), effects on ticarcillin; (**b**), effects on cefepime; (**c**), effects on gentamycin; (**d**), effects on azithromycin; (**e**), effects on novobiocin. FIC Index, Fractional Inhibitory Concentration Index calculated for each tested combination of antibiotic and caffeine according to Odds [26]

Figure S5a

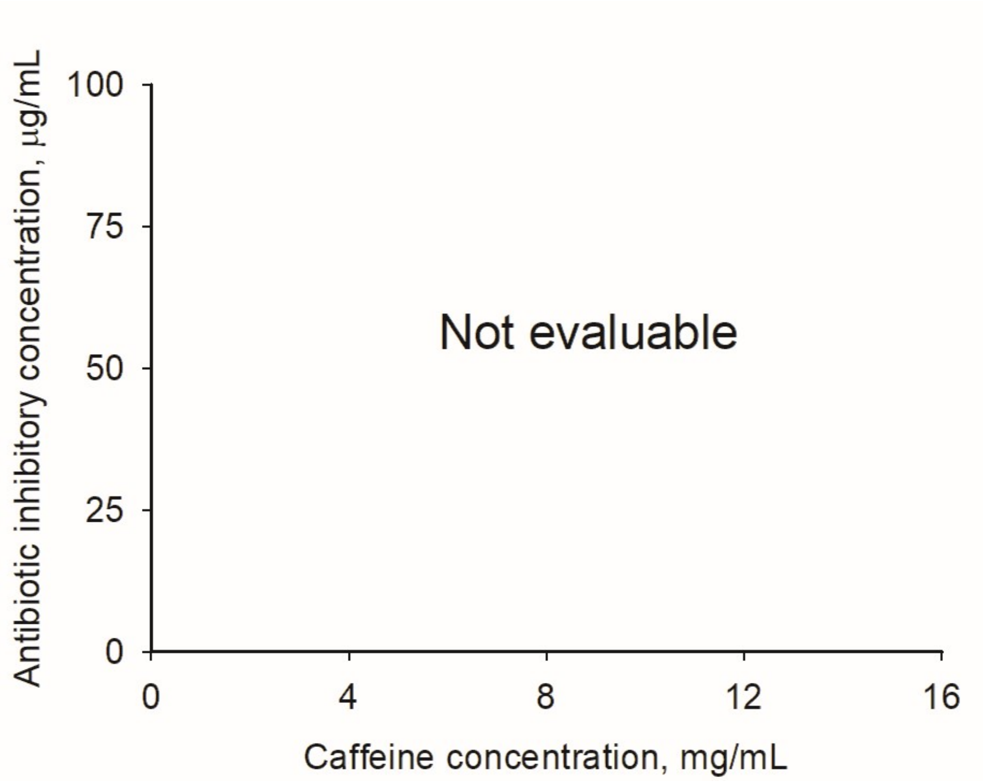

Figure S5b

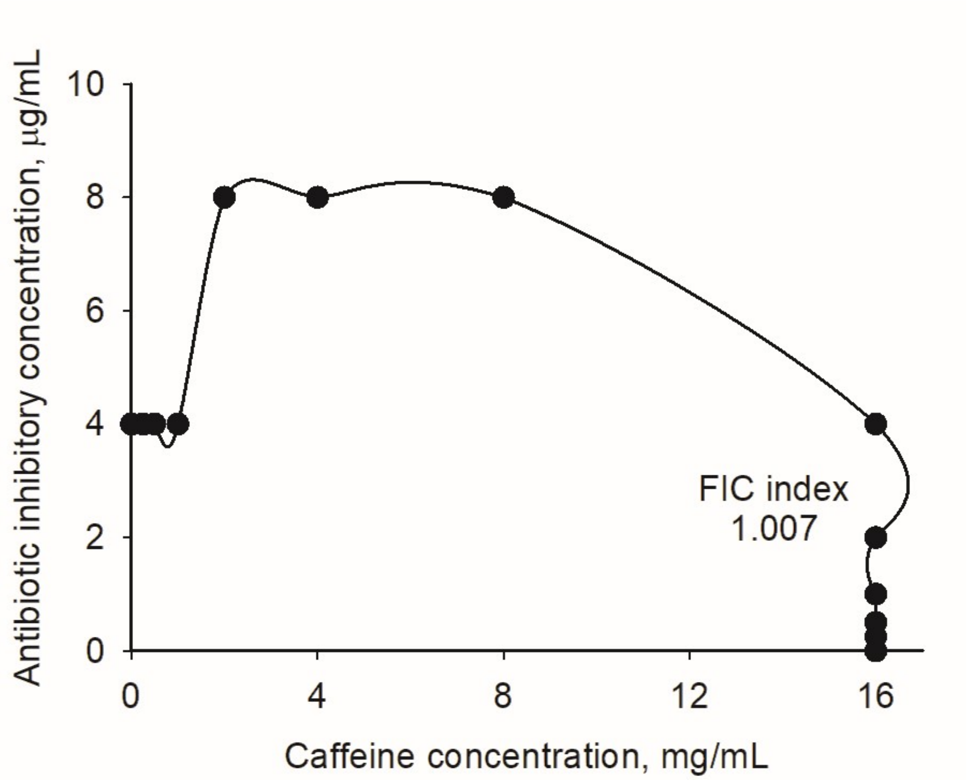

Figure S5c

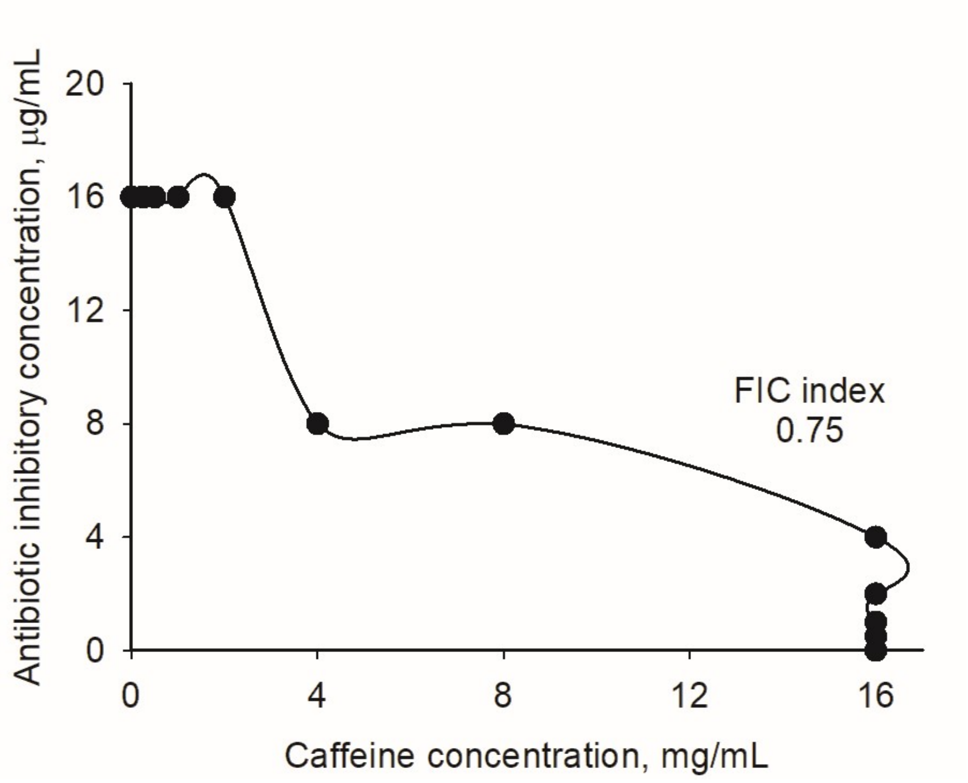

Figure S5d

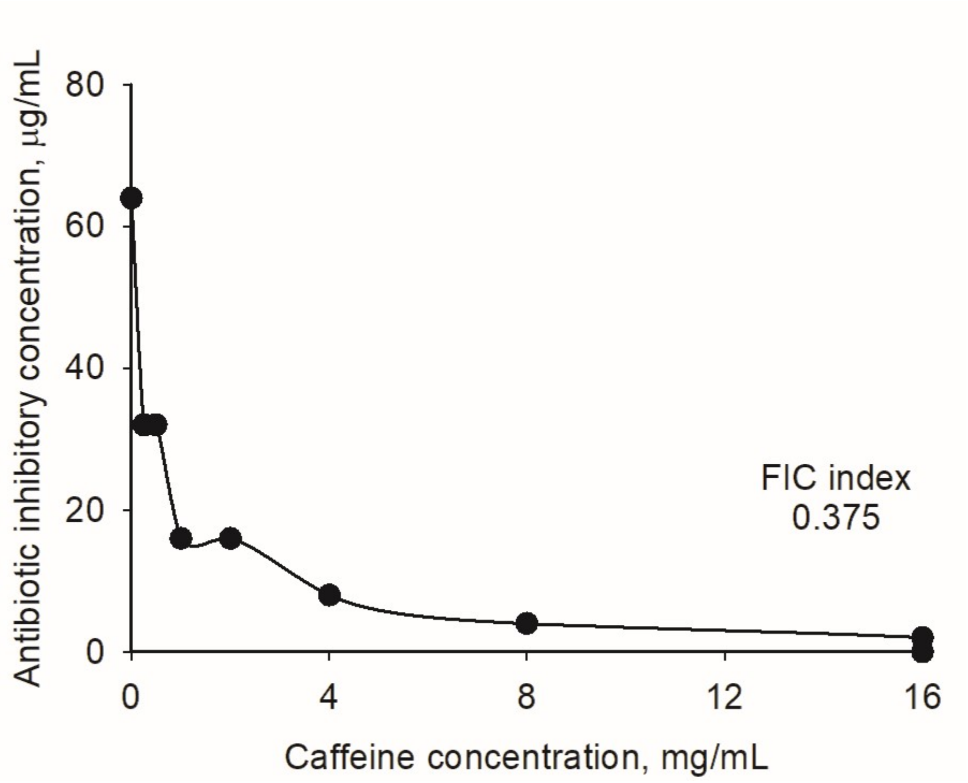

Figure S5e

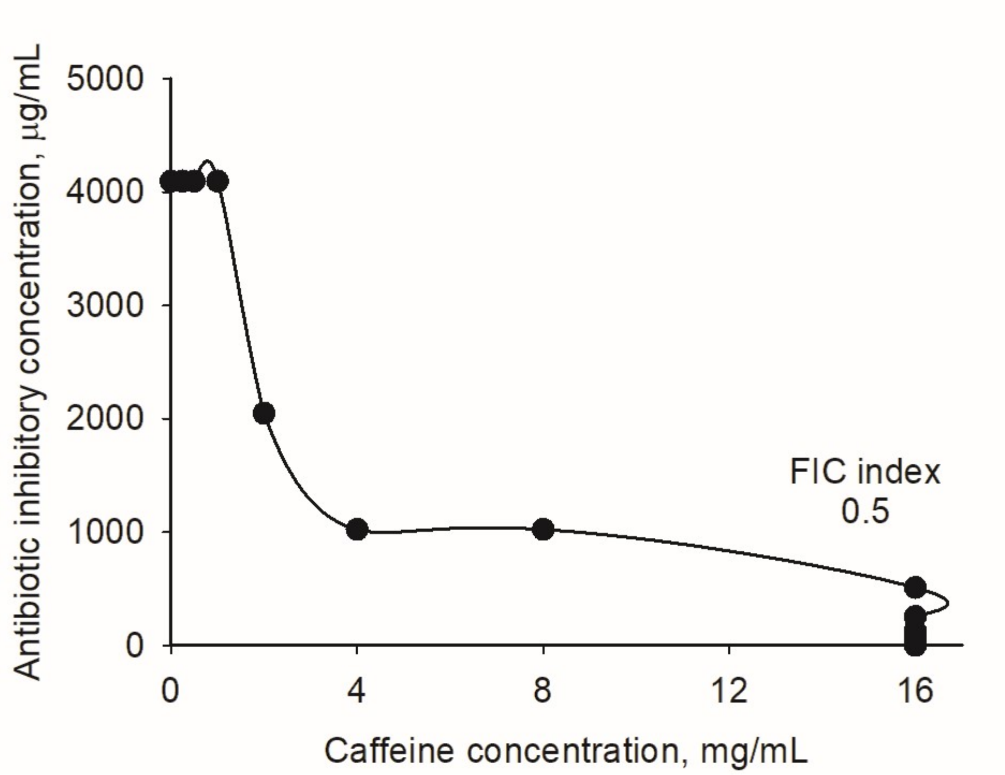

Figure S5. Impact of caffeine on antibacterial activity of antibiotics in *Klebsiella pneumoniae* ATCC 700603 strain using microbroth dilution assay and checkerboard methodology. (**a**), effects on ticarcillin; (**b**), effects on cefepime; (**c**), effects on gentamycin; (**d**), effects on azithromycin; (**e**), effects on novobiocin. FIC Index, Fractional Inhibitory Concentration Index calculated for each tested combination of antibiotic and caffeine according to Odds [26]

Figure S6a

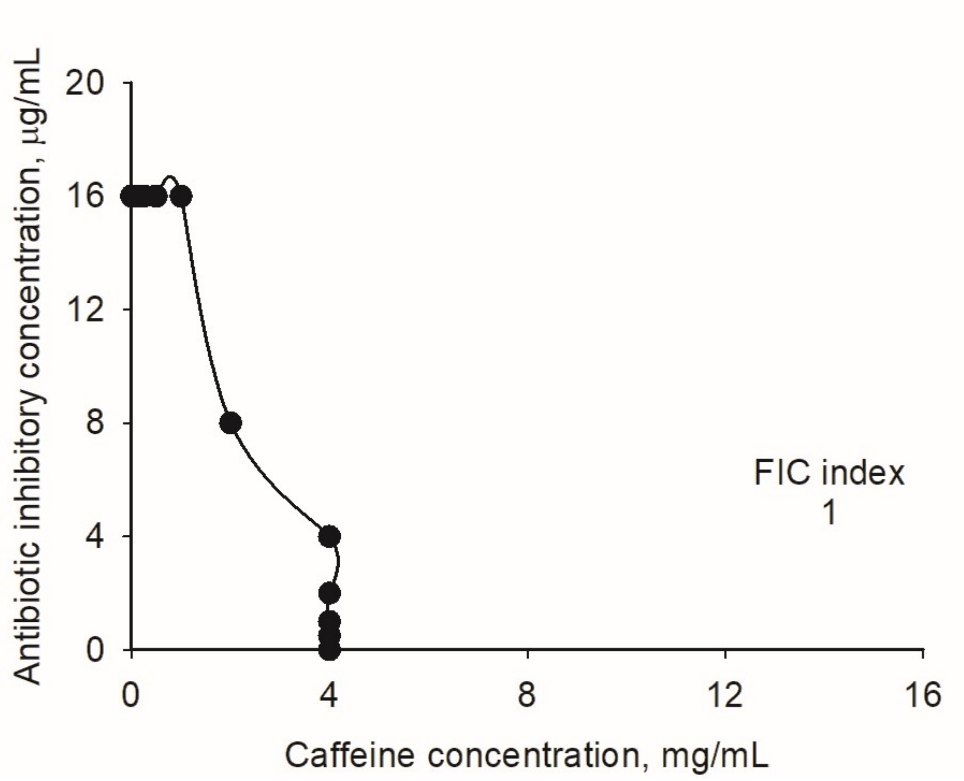

Figure S6b

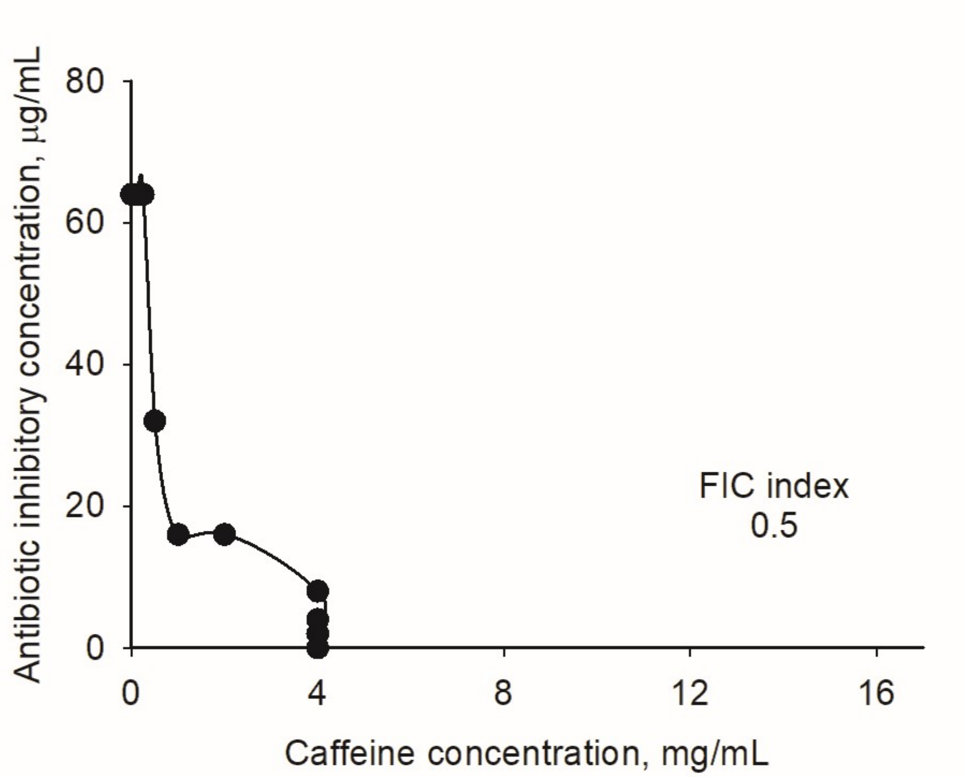

Figure S6c

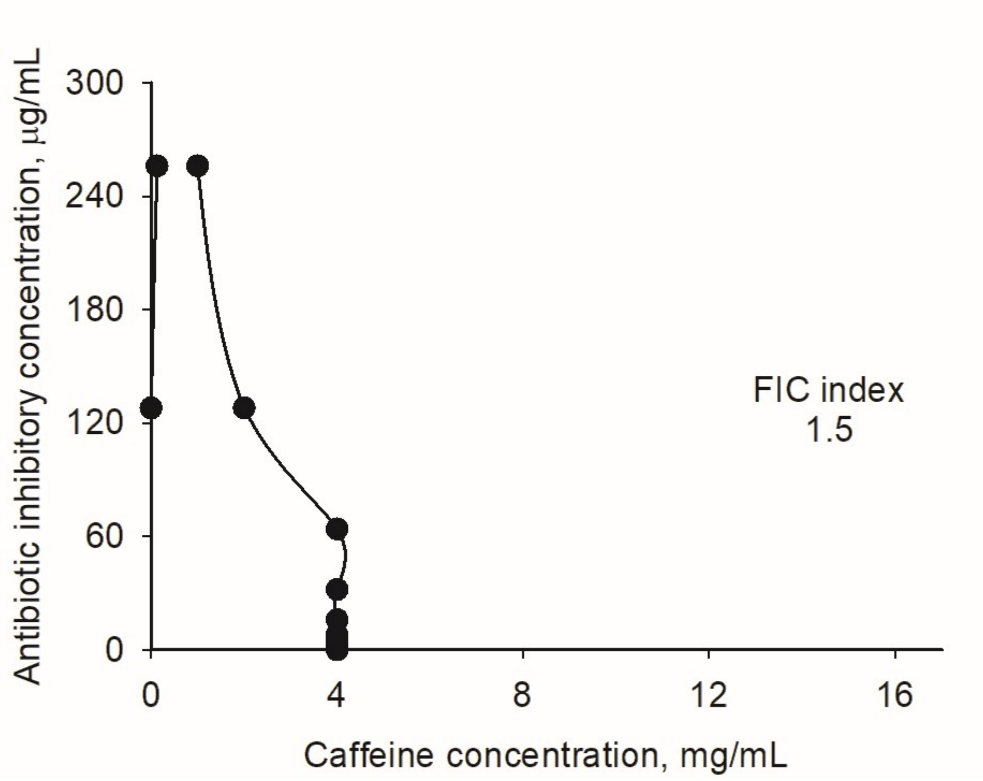

Figure S6d

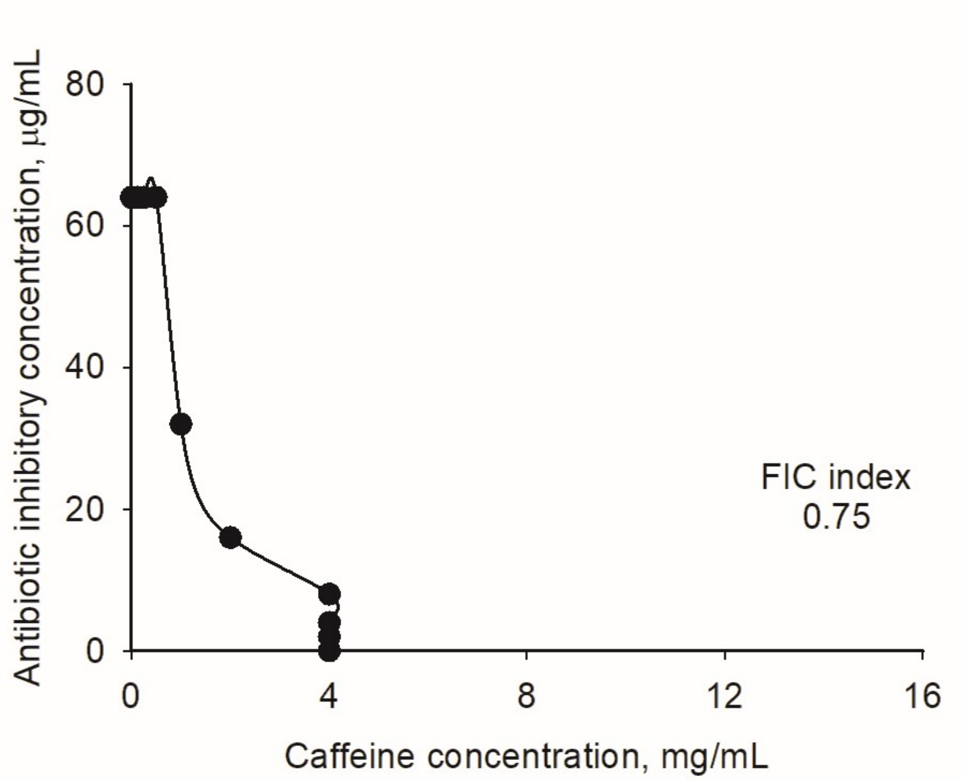

Figure S6e

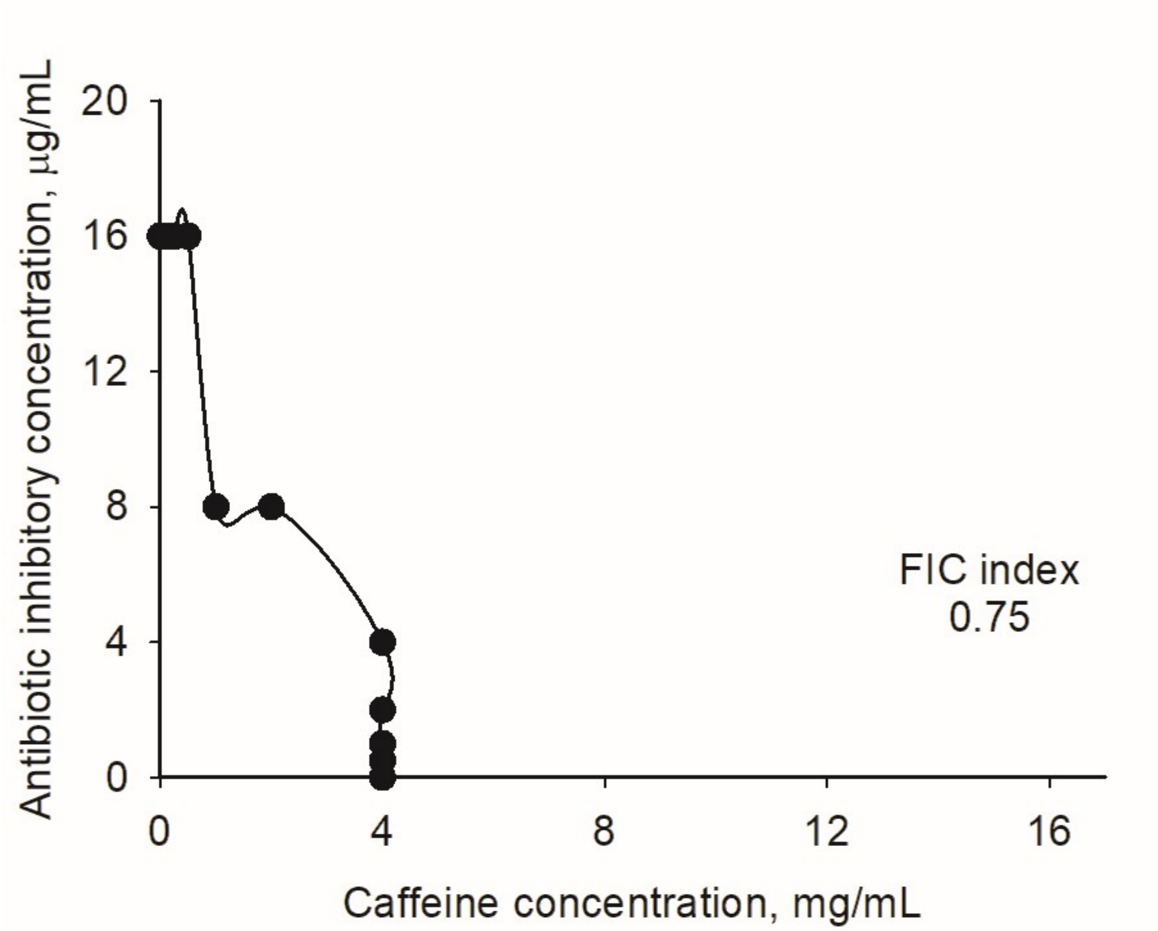

Figure S6. Impact of caffeine on antibacterial activity of antibiotics in *Acinetobacter baumannii* ATCC 19606 strain using microbroth dilution assay and checkerboard methodology. (**a**), effects on ticarcillin; (**b**), effects on cefepime; (**c**), effects on gentamycin; (**d**), effects on azithromycin; (**e**), effects on novobiocin. FIC Index, Fractional Inhibitory Concentration Index calculated for each tested combination of antibiotic and caffeine according to Odds [26]

Figure S7 (a)

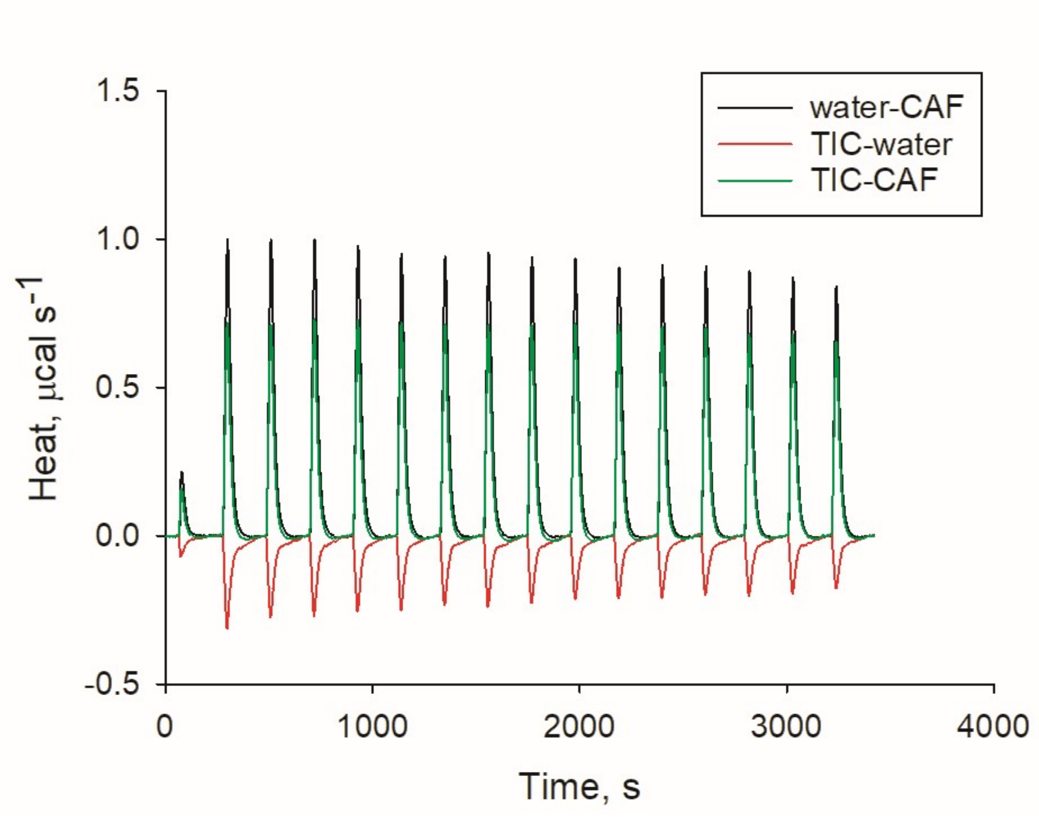

Figure S7 (b)

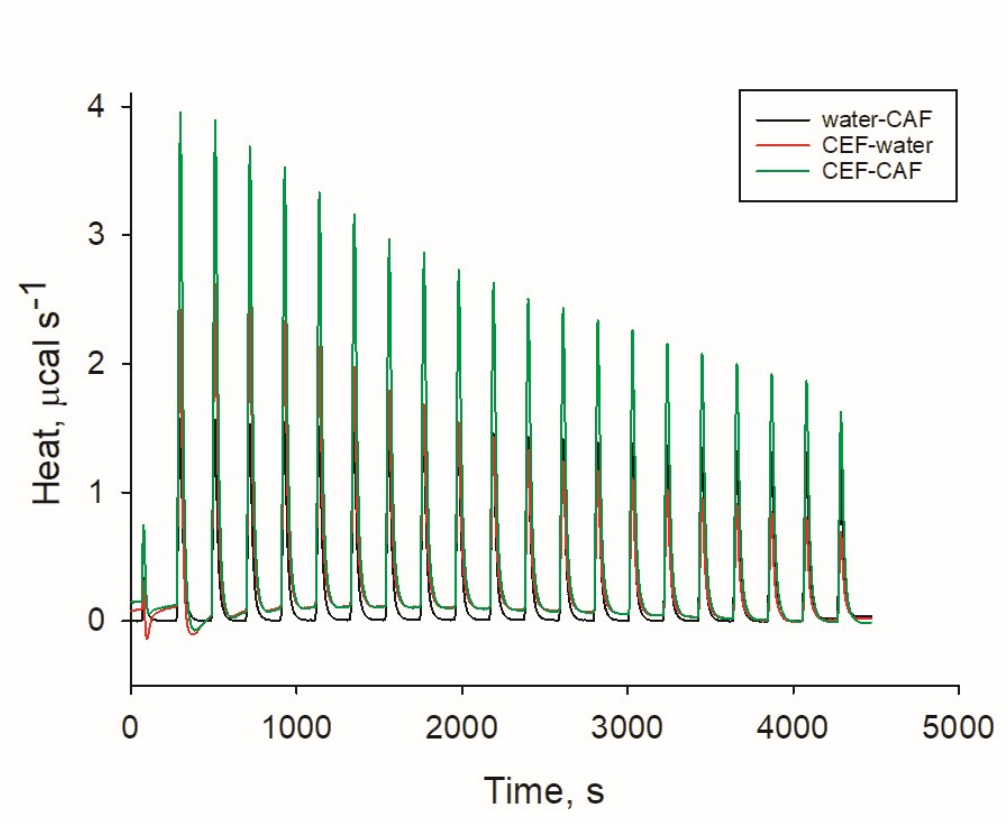

Figure S7 (c)

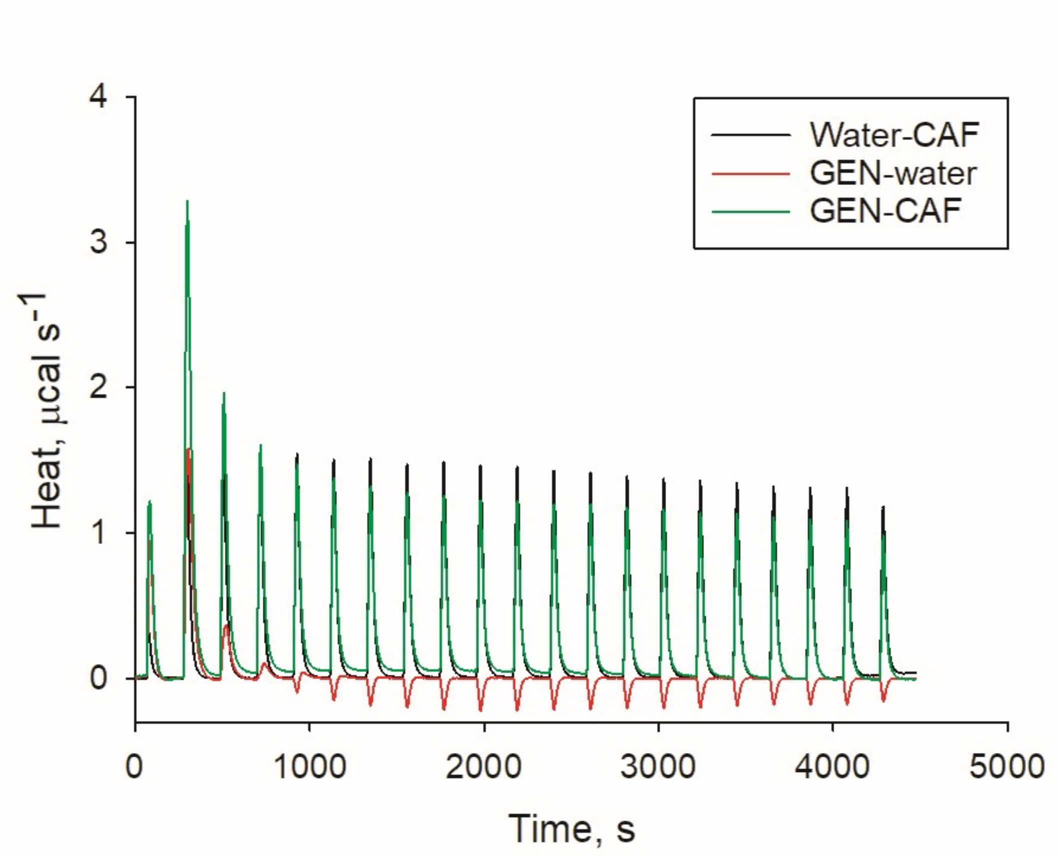

Figure S7 (d)

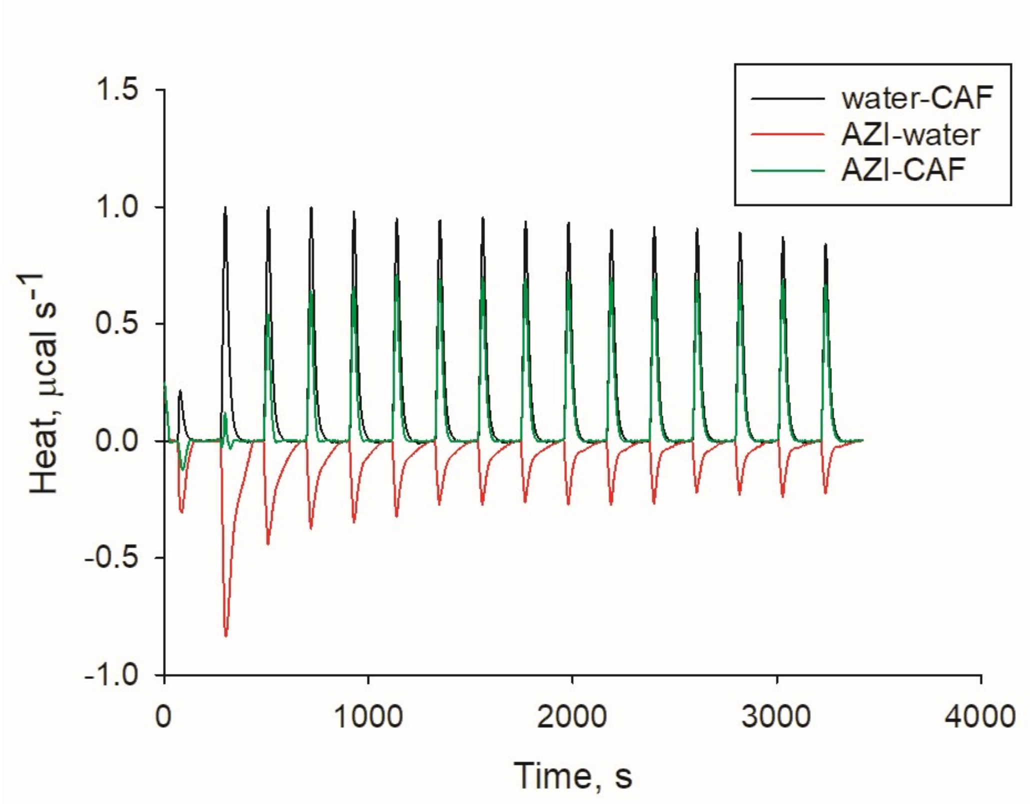

Figure S7 (e)

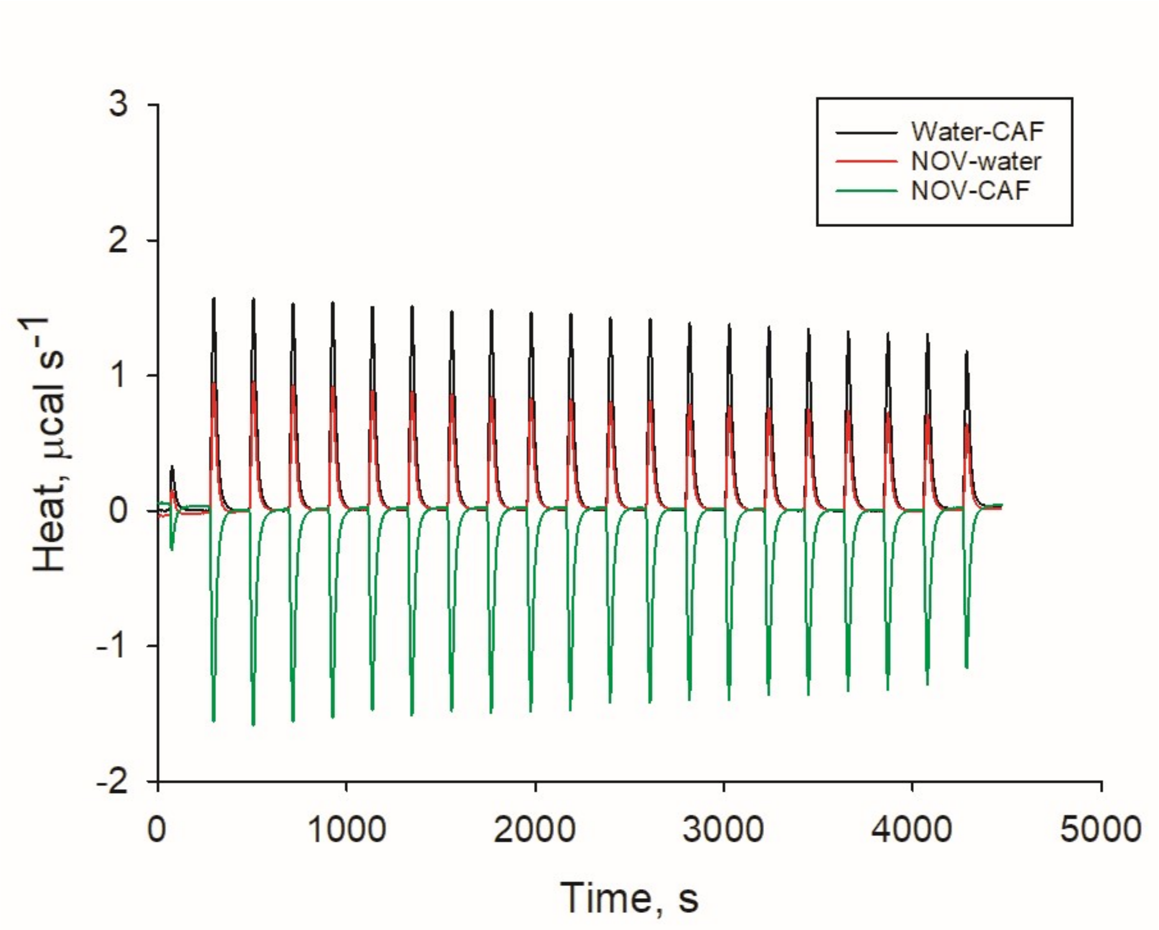

Figure S7. Isothermal titration calorimetry thermograms for analysis of antibiotic-caffeine interactions. (a) ticarcillin (TIC)-caffeine interaction; (b) cefepime (CEF)-caffene interaction; (c), gentamycin (GEN)-caffeine interaction; (d), azithromycin (AZI)-caffeine interaction; (e) novobiocin (NOV)-caffeine interaction. Titration of caffeine with antibiotic is shown in green water with caffeine – in black, and antibiotic with water – in red.

Table S1. Characteristics and antibacterial effects of caffeine toward clinical isolates of *Staphylococcus aureus*

Isolate ID	Methicillin resistance	MIC_{CAF} (mg/mL)	Material	Glyco-peptides	Rifamycines	Quinolones	Fusidic acid	Tetracyclines	Beta-lactams	Aminoglycosides
1	MRSA	16	Bronchus	Wild	Wild	Resistant	Wild	Wild	Modification of PBP	Resistant KAN
2	MRSA	8	Nose	Wild	Wild	Resistant	Wild	Partially resistant[2]	Modification of PBP	Resistant KAN
3	MRSA	8	Bronchus	Wild	Wild	Resistant	Wild	Partially resistant[2]	Modification of PBP	Resistant KAN
4	MRSA	>16	Throat	Wild	Wild	Wild	Wild	Partially resistant[2]	Modification of PBP	Resistant KAN
5	MRSA	8	Blood	Wild	Resistant[1]	Partially resistant	Resistant	Partially resistant[2]	Modification of PBP	Resistant KAN TOB GEN
6	MRSA	16	Ear	Wild	Wild	Wild	Wild	Wild	Modification of PBP	Resistant KAN
7	MRSA	8	bronchus	Wild	Wild	Wild	Wild	Partially resistant[2]	Modification of PBP	Resistant KAN
8	MRSA	8	Nose	Wild	Wild	Wild	Wild	Partially resistant[2]	Modification of PBP	Resistant KAN
9	MRSA	16	Nose	Wild	Wild	Wild	Wild	Partially resistant[2]	Modification of PBP	Resistant KAN TOB GEN
10	MRSA	16	Bronchus	Wild	Resistant[1]	Partially resistant	Wild	Partially resistant[2]	Modification of PBP	Resistant KAN TOB GEN
11	MRSA	16	Nose	Wild	Wild	Wild	Wild	Partially resistant[2]	Modification of PBP	Resistant KAN
12	MRSA	16	Bronchus	Wild	Wild	Resistant	Wild	Partially resistant[2]	Modification of PBP	Resistant KAN
13	MRSA	8	Rectum	Wild	Wild	Partially resistant	Wild	Partially resistant[2]	Modification of PBP	Resistant KAN TOB GEN
14	MRSA	16	Nose	Wild	Wild	Wild	Wild	Partially resistant[2]	Modification of PBP	Resistant KAN
15	MRSA	>16	Nose	Wild	Wild	Wild	Wild	Wild	Modification of PBP	Resistant KAN
16	MRSA	16	Nose	Wild	Resistant[1]	Partially resistant	Resistant	Partially resistant[2]	Modification of PBP	Resistant KAN TOB GEN
17	MRSA	4	Nose	Wild	Resistant[1]	Partially resistant	Resistant	Partially resistant[2]	Modification of PBP	Resistant KAN TOB GEN
18	MRSA	>16	Nose	Wild	Wild	Wild	Wild	Wild	Modification of PBP	Resistant KAN
19	MRSA	8	Nose	Wild	Wild	Resistant	Wild	Wild	Modification of PBP	Resistant KAN
20	MRSA	16	Nose	Wild	Wild	Wild	Wild	Wild	Modification of PBP	Resistant KAN
21	MRSA	16	Nose	Wild	Wild	Resistant	Wild	Wild	Modification of PBP	Resistant KAN
22	MRSA	16	Nose	Wild	Wild	Resistant	Wild	Wild	Modification of PBP	Resistant KAN
23	MRSA	16	Nose	Wild	Wild	Resistant	Wild	Wild	Modification of PBP	Resistant KAN
24	MRSA	16	Bronchus	Wild	Wild	Resistant	Wild	Wild	Modification of PBP	Resistant KAN
25	MRSA	>16	Blood	Wild	Wild	Resistant	Wild	Wild	Modification of PBP	Resistant KAN
26	MRSA	>16	Ulceration	Wild	Wild	Wild	Wild	Wild	Modification of PBP	Resistant KAN
27	MRSA	8	Bronchus	Wild	Resistant[1]	Resistant	Resistant	Partially resistant[2]	Modification of PBP	Resistant KAN TOB GEN
28	MRSA	>16	Ulceration	Wild	Wild	Wild	Wild	Wild	Modification of PBP	Resistant KAN
29	MRSA	16	Abscess	Wild	Wild	Wild	Wild	Wild	Modification of PBP	Resistant KAN
30	MRSA	8	Wound	Wild	Wild	Partially resistant	Wild	Partially resistant[2]	Modification of PBP	Resistant KAN TOB GEN
31	MRSA	4	Blood	Wild	Resistant[1]	Resistant	Resistant	Partially resistant[2]	Modification of PBP	Resistant KAN TOB GEN
32	MRSA	8	aspirate	Wild	Resistant[1]	Partially resistant	Resistant	Partially resistant[2]	Modification of PBP	Resistant KAN TOB GEN
33	MRSA	>16	Urine	Wild	Wild	Wild	Wild	Wild	Modification of PBP	Resistant KAN

Table S1 (continued) Characteristics and antibacterial effects of caffeine toward clinical isolates of *Staphylococcus aureus*

Isolate ID	Methicillin resistance	MIC_{CAF} (mg/mL)	Material	Glyco-peptides	Rifamycines	Quinolones	Fusidic acid	Tetracyclines	Beta-lactams	Aminoglycosides
34	MRSA	16	Wound	Wild	Wild	Resistant	Wild	Wild	Modification of PBP	Resistant KAN
35	MRSA	8	Pus	Wild	Resistant[1]	Partially resistant	Resistant	Partially resistant[2]	Modification of PBP	Resistant KAN TOB GEN
36	MRSA	16	Abscess	Wild	Wild	Partially resistant	Wild	Wild	Modification of PBP	Resistant KAN
37	MRSA	16	Wound	Wild	Wild	Resistant	Wild	Partially resistant[2]	Modification of PBP	Resistant KAN TOB GEN
38	MRSA	>16	Blood	Wild	Wild	Wild	Wild	Wild	Modification of PBP	Resistant KAN
39	MRSA	>16	Wound	Wild	Wild	Resistant	Wild	Wild	Modification of PBP	Resistant KAN
40	MRSA	>16	Wound						Modification of PBP	
41	MRSA	>16	Blood	Wild	Resistant[1]	Partially resistant	Resistant	Partially resistant[2]	Modification of PBP	Resistant KAN TOB GEN
42	MRSA	8	Throat	Wild	Resistant[1]	Resistant	Resistant	Partially resistant[2]	Modification of PBP	Resistant KAN TOB GEN
43	MRSA	16	Abscess	Wild	Wild	Wild	Wild	Wild	Modification of PBP	Resistant KAN
44	MRSA	16	Blood	Wild	Wild	Resistant	Wild	Wild	Modification of PBP	Resistant KAN
45	MRSA	16	Abscess	Wild	Wild	Wild	Wild	Wild	Modification of PBP	Resistant KAN
46	MRSA	>16	Wound	Wild	Wild	Resistant	Wild	Wild	Modification of PBP	Resistant KAN
47	MRSA	16	Blood	Wild	Resistant[1]	Partially resistant	Resistant	Partially resistant[2]	Modification of PBP	Resistant KAN TOB GEN
48	MRSA	>16	Wound	Wild	Resistant[1]	Partially resistant	Resistant	Partially resistant[2]	Modification of PBP	Resistant KAN TOB GEN
49	MSSA	16	Ear	Wild	Wild	Wild	Wild	Wild	Acquired penicillinase	Resistant KAN
50	MSSA	4	Nose	Wild	Wild	Resistant	Wild	Partially resistant[2]	Acquired penicillinase	Resistant KAN
51	MSSA	>16	Nose	Wild	Wild	Wild	Wild	Partially resistant[2]	Acquired penicillinase	Resistant KAN
52	MSSA	16	Skin	Wild	Wild	Wild	Wild	Wild	Acquired penicillinase	Resistant KAN
53	MSSA	16	Navel	Wild	Wild	Wild	Wild	Wild	Acquired penicillinase	Resistant KAN
54	MSSA	16	Nose	Wild	Wild	Wild	Wild	Wild	Acquired penicillinase	Resistant KAN
55	MSSA	>16	Nose	Wild	Wild	Wild	Wild	Wild	Acquired penicillinase	Resistant KAN
56	MSSA	16	Marrow cavity	Wild	Wild	Wild	Wild	Partially resistant[2]	Acquired penicillinase	Resistant KAN
57	MSSA	16	Nose	Wild	Wild	Wild	Wild	Partially resistant[2]	Acquired penicillinase	Resistant KAN
58	MSSA	16	Nose	Wild	Wild	Wild	Wild	Partially resistant[2]	Acquired penicillinase	Resistant KAN
59	MSSA	16	Feces	Wild	Wild	Wild	Wild	Partially resistant[2]	Acquired penicillinase	Resistant KAN
60	MSSA	4	Nose	Wild	Wild	Wild	Wild	Wild	Wild	Resistant KAN
61	MSSA	>16	Skin	Wild	Wild	Wild	Wild	Partially resistant[2]	Acquired penicillinase	Resistant KAN
62	MSSA	>16	Throat	Wild	Wild	Wild	Wild	Wild	Wild	Resistant KAN
63	MSSA	16	Ear	Wild	Wild	Wild	Wild	Wild	Acquired penicillinase	Resistant KAN
64	MSSA	≥16	Nose	Wild	Wild	Wild	Wild	Wild	Acquired penicillinase	Resistant KAN
65	MSSA	16	Throat	Wild	Wild	Wild	Wild	Wild	Acquired penicillinase	Resistant KAN
66	MSSA	16	Throat	Wild	Resistant[1]	Wild	Wild	Partially resistant[2]	Acquired penicillinase	Resistant KAN TOB GEN

Table S1 (continued) Characteristics and antibacterial effects of caffeine toward clinical isolates of *Staphylococcus aureus*

Isolate ID	Methicillin resistance	MIC_{CAF} (mg/mL)	Material	Glyco-peptides	Rifamycines	Quinolones	Fusidic acid	Tetracyclines	Beta-lactams	Aminoglycosides
67	MSSA	16	Skin	Wild	Wild	Wild	Wild	Partially resistant[2]	Acquired penicillinase	Resistant KAN
68	MSSA	16	Bronchus	Wild	Resistant[1]	Partially resistant	Wild	Wild	Acquired penicillinase	Resistant KAN TOB GEN
69	MSSA	>16	Bronchus	Wild	Wild	Resistant	Wild	Wild	Acquired penicillinase	Resistant KAN
70	MSSA	8	Nose	Wild	Wild	Wild	Wild	Wild	Acquired penicillinase	Resistant KAN
71	MSSA	16	Nose	Wild	Wild	Wild	Wild	Partially resistant[2]	Acquired penicillinase	Resistant KAN
72	MSSA	16	Navel	Wild	Wild	Wild	Wild	Wild	Acquired penicillinase	Resistant KAN
73	MSSA	16	Pus	Hetero-VISA	Resistant[1]	Resistant	Resistant	Partially resistant[2]	Wild	Resistant KAN TOB GEN
74	MSSA	8	Ulceration	Wild	Resistant[1]	Resistant	Wild	Partially resistant[2]	Acquired penicillinase	Resistant KAN TOB GEN
75	MSSA	16	Vascular catheter	Wild	Wild	Wild	Wild	Partially resistant[2]	Acquired penicillinase	Resistant KAN
76	MSSA	16	Aspirate	Wild	Wild	Partially resistant	Wild	Partially resistant[2]	Acquired penicillinase	Resistant KAN TOB GEN
77	MSSA	8	Ulceration	Wild	Resistant[1]	Resistant	Wild	Partially resistant[2]	Acquired penicillinase	Resistant KAN TOB GEN
78	MSSA	16	Vascular catheter	Wild	Resistant[1]	Resistant	Resistant	Partially resistant[2]	Wild	Resistant KAN TOB GEN
79	MSSA	8	Wound	Wild	Resistant[1]	Resistant	Wild	Partially resistant[2]	Acquired penicillinase	Resistant KAN TOB GEN
80	MSSA	16	Wound	Wild	Wild	Wild	Wild	Wild	Acquired penicillinase	Resistant KAN
81	MSSA	16	Pus	Wild	Wild	Wild	Wild	Partially resistant[2]	Acquired penicillinase	Resistant KAN
82	MSSA	8	Blood	Wild	Wild	Partially resistant	Wild	Partially resistant[2]	Acquired penicillinase	Resistant KAN TOB GEN
83	MSSA	>16	Blood	Wild	Wild	Wild	Wild	Wild	Acquired penicillinase	Resistant KAN
84	MSSA	8	Blood	Wild	Resistant[1]	Resistant	Resistant	Partially resistant[2]	Wild	Resistant KAN TOB GEN
85	MSSA	>16	Wound	Wild	Wild	Wild	Wild	Partially resistant[2]	Acquired penicillinase	Resistant KAN
86	MSSA	16	Pemphigus	Wild	Wild	Wild	Wild	Wild	Acquired penicillinase	Resistant KAN
87	MSSA	16	Blood	Wild	Wild	Wild	Wild	Wild	Wild	Resistant KAN
88	MSSA	8	Ulceration	Wild	Resistant[1]	Partially resistant	Wild	Partially resistant[2]	Acquired penicillinase	Resistant KAN TOB GEN
89	MSSA	>16	Ulceration	Wild	Wild	Partially resistant	Wild	Partially resistant[2]	Acquired penicillinase	Resistant KAN
90	MSSA	16	Blood	Wild	Wild	Wild	Wild	Wild	Wild	Resistant KAN
91	MSSA	>16	Wound	Wild	Wild	Wild	Wild	Wild	Wild	Resistant KAN
92	MSSA	16	Wound	Wild	Wild	Wild	Wild	Wild	Wild	Resistant KAN
93	MSSA	8	Ulceration	Wild	Wild	Wild	Wild	Partially resistant[2]	Acquired penicillinase	Resistant KAN
94	MSSA	16	Hematoma	Wild	Wild	Wild	Wild	Wild	Wild	Resistant KAN
95	MSSA	16	Post-surgery wound	Wild	Wild	Wild	Wild	Partially resistant[2]	Acquired penicillinase	Resistant KAN
96	MSSA	>16	Ulceration	Wild	Wild	Wild	Wild	Wild	Acquired penicillinase	Resistant KAN
97	MSSA	>16	Blood	Wild	Wild	Wild	Wild	Wild	Acquired penicillinase	Resistant KAN
98	MSSA	16	Blood	Wild	Wild	Wild	Wild	Wild	Acquired penicillinase	Resistant KAN
99	MSSA	>16	Blood	Wild	Wild	Wild	Wild	Wild	Acquired penicillinase	Resistant KAN

[1] high level resistance; [2] tetracycline efflux protein; KAN = kanamycin, TOB = tobramycin, GEN = gentamycin, PBP = penicillin-binding protein
